# Generation of iPSC Lines with Tagged α-Synuclein for Visualization of Endogenous Protein in Human Cellular Models of Neurodegenerative Disorders

**DOI:** 10.1523/ENEURO.0093-25.2025

**Published:** 2025-06-10

**Authors:** Oskar G. Zetterdahl, James A. Crowe, Samira Reyhani, Miriam A. Güra, Ot Labastida-Botey, Aline S. Girard, D. Sean Froese, Henrik Ahlenius, Isaac Canals

**Affiliations:** ^1^Glial and Neuronal Biology Lab, Department of Experimental Medical Science, Lund University, Lund 22184, Sweden; ^2^Stem Cells, Aging and Neurodegeneration Lab, Department of Experimental Medical Science, Lund Stem Cell Center, Lund University, Lund 22184, Sweden; ^3^Division of Metabolism and Children’s Research Center, University Children’s Hospital Zurich, University of Zurich, Zurich 8008, Switzerland

**Keywords:** α-synuclein, genome editing, imaging, induced pluripotent stem cells, tagged protein

## Abstract

α-Synuclein is a synaptic protein that accumulates primarily in synucleinopathies and secondarily in certain lysosomal storage disorders. However, its physiological roles in health and disease are not fully understood. In part, this has been hampered by the inability to visualize α-synuclein and its cellular localization, due to the lack of specific antibodies and faithful reporters. Here, we used CRISPR/Cas9-based genome editing to generate human-induced pluripotent stem cell (iPSC) lines in which the α-synuclein (*SNCA*) gene has been tagged with the short HA peptide either at the N-terminus or C-terminus or with the fluorescent protein mCherry at the C-terminus of the protein. These diverse strategies revealed the C-terminus HA-tag as the best option. C-Terminus HA-tagged α-synuclein had unchanged protein expression and did not generate degradation by-products. Importantly, we show that following differentiation to neurons, the C-terminus HA-tagged iPSC line had unaffected electrophysiological properties and could be used to visualize accumulation of α-synuclein upon inhibition of lysosomal function and under physiological protein levels. It is our expectation that this line and tagging approach will be very useful in further studies examining α-synuclein aggregation and its role in cellular dysfunction and neurodegeneration.

## Graphical abstract

**Figure:**
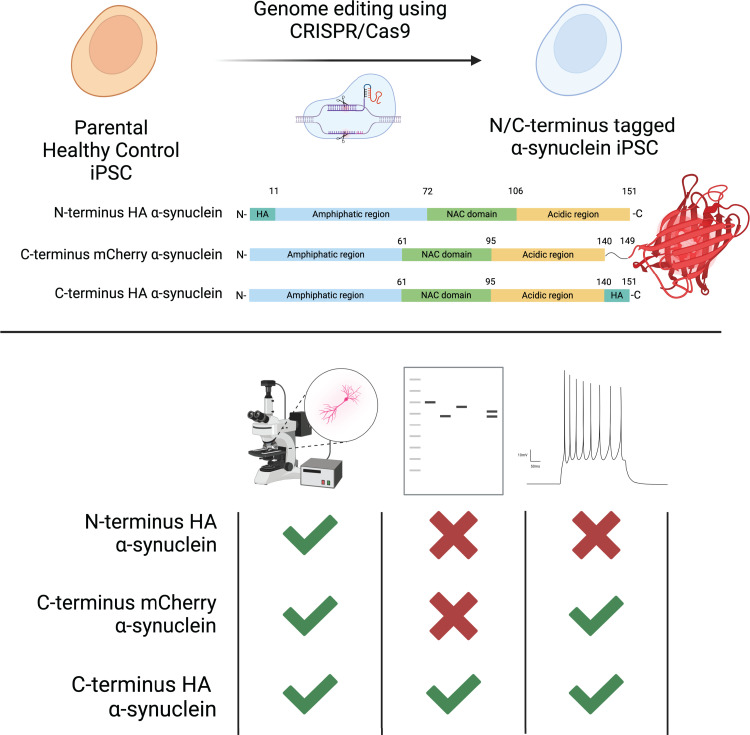
Visual Abstract

## Significance Statement

We present an optimal genome editing strategy for incorporating the short peptide HA at the C-terminus of α-synuclein in human-induced pluripotent stem cells. We also show that this newly generated C-terminus tagged line can be differentiated toward functional neurons to facilitate visualization of the protein and its accumulation upon inhibition of lysosomal function, which will be useful for studying aggregation in models of neurodegenerative diseases.

## Introduction

α-Synuclein is a protein encoded by the *SNCA* gene, which is located in the long arm of chromosome 4 and extends for >100 kb (Lunati et al., 2018). The 140-amino acid α-synuclein protein is mainly expressed in the brain and is restricted to vertebrates (Sulzer and Edwards, 2019). Although the exact role of the protein remains elusive, several studies have shown its capacity to associate with membranes with acidic phospholipid headgroups or high curvature (Davidson et al., 1998; Bussell and Eliezer, 2003; Jao et al., 2004; Georgieva et al., 2008; Bodner et al., 2009). The association of α-synuclein with high curvature membranes (Liu et al., 2021) and its synaptic location suggests an interaction with synaptic vesicles, and a recent study indicated that it is essential for synaptic vesicle clustering, where α-synuclein acts as a double anchor between membranes of different vesicles (Fusco et al., 2016).

When mutated, misfolded, or accumulated, α-synuclein can form insoluble aggregates known as Lewy bodies (Moors et al., 2016; Lunati et al., 2018). These aggregates are one of the hallmarks of Parkinson's disease but can form as a secondary effect in other neurodegenerative disorders caused by dysregulation of the lysosomal system, such as neuronopathic lysosomal storage disorders (Mazzulli et al., 2011; Winder-Rhodes et al., 2012). Most studies on α-synuclein have relied on antibodies, many of which detect other synucleins (Leupold et al., 2022). Other studies on visualization and aggregation have used overexpression paradigms of tagged α-synuclein (Hansen et al., 2011). However, overexpression has been shown to lead to an excessive amount of α-synuclein within the cell, resulting in mitochondrial fragmentation (Kamp et al., 2010). Overexpression of α-synuclein has also been shown to affect synaptic vesicle cycling in induced pluripotent stem cell (iPSC)-derived neurons (White et al., 2024), thus impairing neuronal functionality.

Consequently, there is an urgent need to generate better tools for investigating the specific roles of α-synuclein in health and disease. Facilitating visualization of the protein under normal expression levels, both in fixed and live imaging experiments, could enable specific localization of α-synuclein within neurons and help identify proteins or lipids that specifically interact with α-synuclein.

To visualize α-synuclein under physiological conditions, we generated three different α-synuclein reporter iPSC lines. Using CRISPR/Cas9 genome editing, we tagged the endogenous SNCA gene in a healthy control (HC) iPSC line with either an HA-tag at the N- or C-terminus or with a mCherry-tag at the C-terminus. We then validated these lines to examine any detrimental effects to α-synuclein and in iPSC-derived neurons functional properties. Overall, we identified that a C-terminus HA-tag is the most suitable approach for visualizing α-synuclein and its accumulation under physiological protein levels and without affecting normal neuronal function.

## Materials and Methods

### Fibroblast lines and reprogramming

Human fibroblasts from an 8-year-old male donor were obtained from Coriell Institute (GM08398) and maintained in Dulbecco's modified Eagle's media (DMEM) with GlutaMax (Thermo Fisher Scientific #61965026) supplemented with 10% FBS (Thermo Fisher Scientific #A5256701) at 37°C in humidified air with 5% CO_2_ and passaged upon reaching confluency of ∼80% using 0.25% Trypsin (Thermo Fisher Scientific #25200056). iPSCs were generated through fibroblast reprogramming in accordance with the Stemgent StemRNA 3rd Gen Reprogramming Kit (Reprocell #00-0076).

### iPSC culturing

iPSCs were maintained in mTesR1 media (STEMCELL Technologies #85857) on six-well plates coated with ES-qualified Matrigel (Corning #354277) at a temperature of 37°C in humidified air with 5% CO_2_ with daily media change and passaged with StemPro Accutase Cell Dissociation Reagent (Accutase, Thermo Fisher Scientific #A1110501) upon attaining confluency of ∼80%. After dissociation, cells were centrifuged at 300 × *g* and replated onto fresh Matrigel-coated six–well plates at a density of 2–2.5 × 10^5^ cells per well using media supplemented with 10 µM of ROCK inhibitor (RI; STEMCELL Technologies #72307) in the initial 24 h after replating to enhance cell survival.

### Cloning of sgRNAs into CRISPR-Cas9

All sgRNAs were designed using the Benchling software (www.benchling.com). Their target sequences were adjacent to the start or stop codon of the *SNCA* gene. Oligonucleotides were synthesized by Eurofins Genomics, and 1 µl (100 mM) of each single-stranded oligonucleotide strands, partially complementary to one another, was phosphorylated and annealed in 1 µl of 10× T4 PNK Ligation Buffer (New England Biolabs #B0201SVIAL) and 6.5 µl of ddH20 with 0.5 µl of T4 PNK enzyme (New England Biolabs #B0201S). The reaction was allowed to proceed in a thermocycler at 37°C for 5 min followed by 95°C for 5 min, completing a temperature ramp-down to 25°C at the rate of 5°C/min. The annealed oligonucleotides were then diluted 250 times.

A digestion–ligation reaction was carried out with 100 ng of CRISPR-Cas9 vector pSpCas9(BB)-2A-Puro (PX459) V2.0 or pSpCas9n(BB)-2A-Puro (PX462) V2.0, both gifts from Feng Zhang (Addgene plasmid #62988 and #62987, respectively; Ran et al., 2013), 2 µl of the diluted oligonucleotide duplex in a mixture containing 2 µl 10× FastDigest Buffer (Thermo Fisher Scientific #B64), 1 µl FastDigest BpiI (Thermo Fisher Scientific #FD1014), 0.5 µl T7 DNA ligase (New England Biolabs #M0318S), 1 µl DTT (10 mM, Thermo Fisher Scientific #P2325), and 1 µl ATP (10 mM, New England Biolabs #P0756S), with ddH_2_0 added to make up a final reaction volume of 20 µl. The reaction mixture was then incubated in a thermocycler at 37°C for 5 min followed by 23°C for 5 min for six cycles. Following this, 11 µl of the ligation reaction was treated with 1 µl of PlasmidSafe Exonuclease (Nordic Biolabs #E3101K) in 1.5 µl of 10× PlasmidSafe Buffer (Nordic Biolabs #E3101K) containing 1.5 µl of 10 mM ATP, and the reaction was incubated at 37°C for 30 min. Sequences of sgRNAs used can be found in Extended Data Fig. 1-1.

### Homology-directed repair donor DNA

Single-strand donor oligonucleotides (ssODNs) for the HA tags were designed to contain 45–48 nt homology arms on each side of the sgRNA target sequence, a silent mutation in the PAM sequence, the HA-tag sequence (27 nt), phosphothiorate bonds at both ends of the donor strands, and were synthesized by Integrated DNA Technologies. The mCherry donor DNA consisted of the mCherry sequence (711 nt), a linker sequence upstream of the mCherry cDNA, and homology arms of 500 nt on each side followed by the target sequence for the sgRNA115. The donor sequence was synthesized and cloned into the pUC57 plasmid (GenScript). Sequences of all donor DNAs can be found in Extended Data Fig. 1-2.

### Genome editing by transfection of CRISPR-Cas9 vectors

The day before transfection (Day −1), iPSCs were plated at 2.5 × 10^5^ to 3 × 10^5^, into Matrigel-coated six–well plates and incubated overnight. On Day 0, medium was replaced with mTeSR1 with 10 µM RI. A mix of 100 µl OptiMEM (Thermo Fisher Scientific #31-985-062) and 6 µl Lipofectamine Stem Transfection Reagent (Thermo Fisher Scientific #15781918) was prepared alongside another mix of 100 µl OptiMEM, 0,5 µg of the donor DNA, and 1.5 µg of the CRISPR-Cas9 vectors. The contents of the two tubes were then mixed and incubated at room temperature for 10 min prior to being added to the cells in a dropwise manner.

On Days 1 and 2, media were changed to mTeSR1 containing 10 µM RI and puromycin (1.25 µg/ml, Thermo Fisher Scientific #A1113803). On Day 3, media were changed to mTeSR1 media containing 10 µM RI. On Day 4, media were replaced with mTeSR1 media containing 5 µM RI. From Day 5 onward, cells were fed daily with mTeSR1 media until they reached ∼50% confluency. Cells were then dissociated with Accutase and replated at a density of 50-200 cells per well onto Matrigel-coated six–well plates containing mTeSR1 media supplemented with CloneR2 (1:10, STEMCELL Technologies #100-0691). Media were changed after 48 h and again supplemented with CloneR2 (1:10) for another 48 h. Cells were then maintained in mTeSR1 with daily media changes until isolated colonies were ∼1 mm in diameter.

Individual colonies were picked and plated into Matrigel-coated 48–well plates, in mTeSR1 media supplemented with 10 µM RI and penicillin/streptomycin (P/S; 100 µg/ml, Thermo Fisher Scientific #15140122). Media were changed after 48 h, and P/S was added to the mTeSR1 media. Cells were maintained with P/S for three more daily feeds with mTeSR1, followed by expansion until 80% confluency. Cells were then dissociated and collected using Accutase and were plated into 12-well Matrigel–coated plates. Once they reached 70–80% confluency, they were dissociated using Accutase, and 80% of the cells were preserved in CryoStor CS10 (STEMCELL Technologies #07930) at −150°C, and the remaining 20% was used for DNA extraction and further analysis.

### Sequencing

DNA was extracted using the DNeasy Blood and Tissue kit (QIAGEN #69504) following manufacturer's instructions. All sequencing was done through automated Sanger sequencing (Eurofins Genomics and Microsynth).

### Immunocytochemistry

Cells were washed three times with Dulbecco's phosphate-buffered saline (DPBS) prior to being fixed in 4% paraformaldehyde (PFA) for 15 min at room temperature and washed three times with DPBS (Thermo Fisher Scientific #14190144). Fixed cells were permeabilized and blocked with DPBS containing 0.025–0.25% Triton X-100 (Thermo Fisher Scientific #327371000) and 5% normal donkey serum (Sigma-Aldrich #S30-100ML) for 1 h at room temperature. Primary antibodies were incubated overnight at 4°C. The following day, cells were washed twice for 5 min with Triton X-100-supplemented DPBS and blocked again for 5 min. Cells were incubated with appropriate secondary antibodies and Hoechst 33342 (2 µg/ml, Invitrogen #H3570) for 2 h at room temperature and then washed twice with DPBS for 5 min and once with ddH_2_O and mounted with PVA:DABCO mounting media. All antibodies and working dilutions can be found in Extended Data Fig. 1-3.

### RNA isolation, cDNA synthesis, and RT-qPCR

RNA extractions were performed directly from plated cells using the RNeasy kit (QIAGEN #74104) following the manufacturer's instructions. A 1 µg of the RNA was used to synthesize cDNA using the QuantaBio qScript cDNA Synthesis Kit (Avantor Sciences #101414-098), following manufacturer's instructions. TaqMan gene expression assays were used for the RT-qPCR reactions, with 50 ng of cDNA in each reaction well. RT-qPCR for 45 amplification cycles was performed using the Bio-Rad iQ5 Multicolor RT-qPCR Detection System with Bio-Rad iQ5 software and Bio-Rad QuantStudio 7 with Design and Analysis 2.6.0 software. All TaqMan assays used can be found in Extended Data Fig. 1-4.

### Karyotyping

iPSCs at 70% confluency (∼9 × 10^6^ cells) were treated with KaryoMAX Colcemid (Thermo Fisher Scientific #15212012) in mTeSR1, at a final concentration of 20 ng/ml for 45 min in humidified air at 37°C with 5% CO_2_. Cells were washed twice with DPBS prior being dissociated with Accutase, centrifuged at 300 × *g* for 5 min, washed with DPBS, and once again centrifuged again for 5 min. The supernatant was aspirated, and while vortexing, 2 ml of 37°C hypotonic solution (KaryoMAX KCl 0.075 M, Thermo Fisher Scientific #10575090) was added drop by drop, followed by a faster addition of another 8 ml. The solution was incubated at 37°C for 10 min followed by the drop-by-drop addition of 1 ml of −20°C Carnoy fixative (3:1 methanol and acetic acid, Sigma-Aldrich #322415 and Sigma-Aldrich #695092, respectively). The solution was centrifuged at 300 × *g* for 10 min after which the supernatant was aspirated. The pellet was resuspended with 2 ml of −20°C Carnoy fixative added drop by drop while vortexing, followed by the faster addition of another 8 ml of Carnoy fixative. Tubes were sealed and kept at −20°C. G-Band karyotype analysis was performed by the karyotyping service at Hospital Sant Joan de Déu (Barcelona, Spain).

### Trilineage differentiation

iPSCs were plated onto Matrigel-coated 24–well plates with mTeSR1 media supplemented with 10 µM of RI at a density of 1 × 10^5^ cells for endoderm and ectoderm fates and 5 × 10^4^ cells for mesoderm fate. Differentiation toward the three germ layers was directed using STEMdiff Trilineage Differentiation Kit (STEMCELL Technologies #05230) for 7 d, before being fixed with 4% PFA and stained for markers of the corresponding germ layer.

### Lentiviral production

Lentiviral vectors used were FUW-M2-rtTA (reverse tetracycline-controlled transactivator), a gift from Rudolf Jaenisch [Addgene, #20342 (Hockemeyer et al., 2008)]; tet-O-Ngn2-puro, a gift from Marius Wernig [Addgene, #52047 (Zhang et al., 2013)]; and tetO-Sox9-Puro and tetO-Nfib-Hygro, both gifts from Henrik Ahlenius [Addgene, #117269 and #117271, respectively (Canals et al., 2018)]. All lentiviruses were produced in HEK 293T cells using packaging plasmids pMDLg/pRRE (Addgene, #12251), pMD2.G (Addgene, #12259), and pRSV-Rev (Addgene, #12253) which were gifts from Didier Trono (Dull et al., 1998). Briefly, HEK 293T cells were cotransfected with the packaging plasmids and one lentivector, ∼44 h following transfection viruses were pelleted by centrifugation (20,000 rpm at 4°C for 2 h), 100 µl of DMEM added, incubated ON at 4°C, resuspended, aliquoted, and frozen at −80°C for long-term storage.

### Differentiation toward induced astrocytes (iAs), induced neurons (iN), and cocultures

The generation of astrocytes, neurons, and cocultures was performed following previously published protocols (Zhang et al., 2013; Canals et al., 2018; Quist et al., 2021). Briefly, iPSCs at ∼80% confluency were dissociated with Accutase (Day −2) and 4 × 10^5^ cells (iAs) or 3 × 10^5^ cells (iNs) were plated on Matrigel-coated six–well plates with mTeSR1 supplemented with 10 µM RI. The following day (Day −1), media were replaced with fresh mTeSR1 media, and rtTA, Sox9, and Nfib (iAs) or rtTA and Ngn2 (iN) lentivirus were added to each well. On Day 0, media were replaced with fresh mTeSR1 media containing doxycycline (2.5 µg/ml, Sigma-Aldrich #D9891), which was kept in the media throughout experiments. For iAs, on Days 1 and 2, DMEM/F12 GlutaMax (Thermo Fisher Scientific #31331093) with 10% FBS and 1% N-2 supplement (Thermo Fisher Scientific #17502001) was used. Between Days 3 and 5, media was gradually changed to FGF medium consisting of Neurobasal with 2% B-27 Supplement (Thermo Fisher Scientific #17504044), 1% NEAA (Thermo Fisher Scientific #11140050), 1% GlutaMax (Thermo Fisher Scientific #35050061), 1% FBS, 8 ng/ml FGF (Thermo Fisher Scientific #100-18B), 5 ng/ml CNTF (Thermo Fisher Scientific #AF-450-13), and 10 ng/ml BMP4 (Thermo Fisher Scientific #120-05ET). Three days of Puromycin (2.5 µg/ml) and five days of hygromycin (200 µg/ml, Thermo Fisher Scientific #10687010) selection was performed. For iN, from Day 1, BrainPhys media (STEMCELL Technologies #05790) with 0.5% N-2 supplement and 1% B2-7 supplement were used and 72 h of Puromycin (2.5 µg/ml) selection was performed. From Day 5, media were supplemented with NT3 (10 ng/ml, Thermo Fisher Scientific #450-03) and BDNF (10 ng/ml, Thermo Fisher Scientific #450-02).

To establish cocultures of iAs and iN, on Day 7, iAs were dissociated with Accutase supplemented with DNase I (100 units/ml, Sigma-Aldrich #11284932001) over 10 min and pelleted for 5 min at 300 × *g*. iN were dissociated for 10 min in Accutase, followed by manual resuspension in the Accutase, 5 more min of incubation, pelleted for 5 min at 300 × *g*, and strained through a 40 µm filter to discard aggregates. Then 3.9 × 10^4^ iAs were plated together with 1.11 × 10^5^ iN on PEI + LAM521-coated 24-well µ–plates (ibidi #82426). From here, 50% iN media and 50% iAs maturation media were used with half media change every 2–3 d. From Day 9 on, FGF medium was changed to maturation medium consisting of 1:1 DMEM/F12 and Neurobasal, 1% N-2 supplement, 1% sodium pyruvate (Thermo Fisher Scientific #11360070), 10 µg/ml NAC (Sigma-Aldrich #A8199), 10 ng/ml hbEGF (Sigma-Aldrich #E4643), 10 ng/ml CNTF, 10 ng/ml BMP4, and 500 µg/ml dbcAMP (Sigma-Aldrich #D0627). From Day 9 to 21, 5-fluoro-2′-deoxyuridine (20 µM, Sigma-Aldrich #F0503) was added to the media to inhibit cell proliferation.

### Western blot

Cell pellets were resuspended in RIPA buffer (Thermo Fisher Scientific # 9900) with 1× protease and phosphatase inhibitor cocktail (Thermo Fisher Scientific #78440) and lysed through sonication for 2 min (10 s on/off intervals), followed by centrifugation at 20,000 × *g* at 4°C for 10 min. Protein concentration was determined using a BCA assay (Thermo Fisher Scientific #23227). Protein lysates were diluted with lysis buffer and 1× Laemmli buffer (Bio-Rad Laboratories #161-0747) with a final concentration of 5% β-mercaptoethanol (Sigma-Aldrich M6250) and incubated at 96°C for 5 min with subsequent cooling on ice. Samples for the SDS-PAGE were loaded on precasted 4–20% gradient gels (Invitrogen #XP04200BOX) and ran at 140 V until the sample front reached the end of the gel. Proteins were transferred onto a 0.2 µm PVDF membrane (Bio-Rad Laboratories #1620177) using the Trans-Blot Turbo Transfer System (Bio-Rad Laboratories) and 1× transfer buffer (10× stock: 25 mM TrisBase, 192 mM glycine) with 20% methanol. Successful transfer was confirmed by staining with Ponceau S solution (Sigma-Aldrich # P7170-1L). Blocking was performed with 5% milk powder (w/v, Millipore Sigma #70166-500G) in 1× Tris-buffered saline (20 mM TrisBase, 150 mM NaCl) with 0.2% Tween 20 (TBS-T; v/v, Sigma-Aldrich #P1379-100ML) and for at least 60 min at ambient temperature. Incubation with primary antibodies was performed overnight at 4°C. After washing with TBS-T, the membranes were incubated with the corresponding secondary antibodies (conjugated with horseradish peroxidase) for 1 h at room temperature. Chemiluminescent signals were acquired on a ChemiDocTM XRS (Bio-Rad Laboratories) using Clarity MaxTM Western ECL Substrate (Bio-Rad Laboratories #1705062). Membranes were stripped following the manufacturer's instructions using Restore Western Blot Stripping Buffer (Thermo Fisher Scientific #21059). Unless otherwise stated, chemicals were purchased from Sigma-Aldrich. All antibodies and working dilutions can be found in Extended Data Fig. 1-3.

### Electrophysiological recordings

Cocultures of iAs and iNs were grown on glass coverslips and transferred to the recording chamber for in vitro recordings. The coverslips were continuously perfused with carbogen (95% O_2_, 5% CO_2_)-gassed artificial cerebrospinal fluid (in mM:119.0 NaCl, 2.5 KCl, 1.3 MgSO_4_, 2.5 CaCl_2_, 26.0 NaHCO_3_, 1.25 NaH_2_PO_4_, and 11.0 glucose), pH ∼7.4, at 34°C. Neurons were visualized using a fixed-stage Olympus Microscope (BX51WI) and a 40× water immersion objective. Patch pipettes were pulled from 1.5 mm borosilicate-glass pipettes with filament using a Sutter P-1000 puller (Sutter Instrument), with a typical resistance of 5–7 MΩ when filled with internal solution (in mM): 135.0 KMeSO_4_, 5.0 KCl, 0.5 CaCl_2_, 5.0 HEPES, 5.0 egtazic acid (EGTA), 2.0 Mg-ATP, and 0.5 Na-GTP, pH 7.25–7.30 (270–280 mOsm).

Recordings were performed using a HEKA EPC10 amplifier (HEKA Elektronik) and sampled at 10 kHz. The PatchMaster software was used for data acquisition. Pipette current was corrected online before giga-seal formation, while fast capacitive currents were compensated for during cell-attached configuration. After the formation of a GΩ seal, the patch was ruptured giving direct access to the intracellular compartment. Access resistance of patched neurons was monitored throughout the experiment using a −5 or −10 mV test pulse from the holding potential of −70 mV. Input resistance (Ri) was measured from the steady-state current during the pulse. To minimize recording errors, series resistance (Rs) was compensated to 60–80%. Membrane capacitance (Cm) was calculated from capacitive currents using the relation Cm = τ/Rs. Resting membrane potential (RMP) and spontaneous firing of action potentials (APs) were measured immediately after opening the cell in the current-clamp mode without injecting current.

APs were evoked using a 400 ms depolarizing current steps (10 pA increments) starting from 0 pA. AP features were measured on the first observed spike evoked by the minimum current necessary to evoke a spike (i.e., rheobase). The AP threshold was determined as the voltage at which dV/dt first exceeded 10 mV/ms. The AP peak amplitude was measured from the threshold to the AP peak. The AP half-width was measured as the time at half-maximal amplitude. The number of APs generated during each current step was counted, and the firing frequency was calculated by dividing the number of spikes by the duration of the firing period. The maximum firing frequency was defined as the highest rate achieved at any depolarizing step.

Spontaneous synaptic activity was recorded in whole-cell voltage–clamp mode at −70 mV for up to 3 min. Spontaneous postsynaptic currents (sPSCs) were detected and analyzed off-line using Igor Pro (WaveMetrics) with the NeuroMatic package, and each event was checked and identified as an sPSC manually.

### Drug treatment of cocultures

Three-week-old cocultures of iAs and iNs were treated with a final concentration of 25 µM GCase inhibitor conduritol-b-epoxide (CBE, Tocris Bioscience #7056). Treatment was done along with every medium change three times per week, and cells were fixed with PFA 4% after 3 weeks of treatment.

Six-week-old cocultures were treated with a final concentration of 200 nM of autophagy inhibitor Bafilomycin A1 (BFA1, Sigma-Aldrich #196000) for 48 h before fixation of samples with PFA 4%.

### Quantification of HA intensity in drug-treated cocultures

HA intensity of BFA1- and CBE-treated and untreated cocultures derived from the C-terminus HA-tag line was quantified in a total of 12 representative images derived from three independent differentiations. Images were acquired with the exact same settings between conditions and within each experiment, and pixel average intensity measurements were performed using the threshold “Moments” function (Fiji) to account only for positive pixels within each image. Quantifications are shown normalized within each experiment toward the untreated condition.

### Statistics

**Figure 1.**
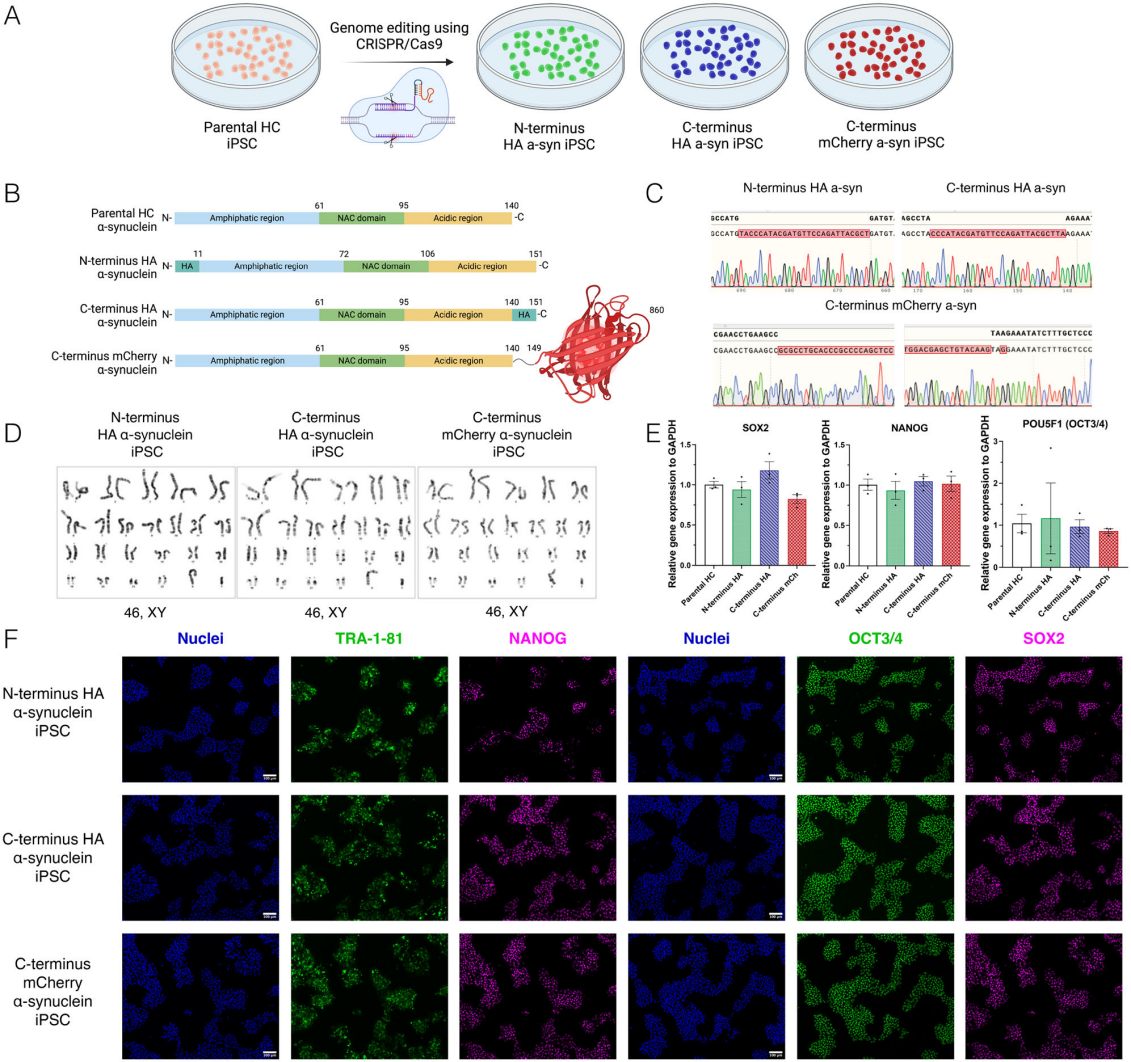
Generation and characterization of different SNCA-tagged iPSC lines using CRISPR/Cas9 genome editing. ***A***, Schematic representation of the generation of tagged lines through CRISPR/Cas9-based genome editing. ***B***, Schematic representation of untagged and tagged α-synuclein proteins, showing the different domains of the protein, the amphipathic region, the non-amyloid component (NAC) domain, and the acidic region, as well as the location of the introduced tags. ***C***, Chromatogram analyses from the tagged lines with the corresponding tag sequence in the corresponding region the SNCA gene. ***D***, Representative G-band karyotyping analyses of the tagged iPSC lines. ***E***, Relative expression of SOX2, NANOG, and POU5F1 in the tagged lines normalized to GAPDH expression and relative to the HC parental line. Bar graphs show the mean ± SEM, from three independent experiments (one-way ANOVA). ***F***, Representative images of iPSCs for each tagged line, positive for markers of undifferentiated state, TRA-1-81, NANOG, OCT3/4, and SOX2. Scale bar, 100 µm. Extended Data Figures 1-1–1-9 are supporting Figure 1.

One-way ANOVA was conducted to compare the relative gene expression of the markers of undifferentiated state (Fig. 1*E*). Gaussian distribution and equal standard deviation assumed. A significance level of *α* = 0.05 was used. Results are presented as mean ± SEM.

One-way ANOVA was used to compare the means of the electrophysiological parameters across the three groups of neurons. Assumptions of normality and homogeneity of variances were verified prior to analysis. A significance level of *α* = 0.05 was used. Results are presented as mean ± SEM, with sample sizes (*n*) indicated.

One-tailed ratio paired *t* test was performed to analyze the increase in HA intensity between BFA1- or CBE-treated and untreated cells, all derived from the C-terminus HA line. A significance level of *α* = 0.05 was used. Results are presented as mean ± SEM, with sample sizes *n* = 3.

## Results

### Generation of three different *SNCA*-reporter iPSC lines using CRISPR/Cas9 genome editing

To facilitate studies on α-synuclein biology and aggregation in synucleopathies and related disorders, we first generated a HC iPSC line through mRNA-based reprogramming of apparently HC fibroblasts (Extended Data Fig. 1-5*A*). This line had no karyotype abnormalities (Extended Data Fig. 1-5*B*); displayed typical iPSC morphology; had high expression of the undifferentiated state markers TRA-1-81, NANOG, OCT3/4, and SOX2 on a protein level (Extended Data Fig. 1-5*C*); and had the capacity to differentiate toward endoderm, ectoderm, and mesoderm (Extended Data Fig. 1-5*D*).

We next utilized the newly generated parental HC iPSC line with CRISPR/Cas9 to introduce different reporter tags to the endogenous SNCA gene (Fig. 1*A*). We designed three different α-synuclein tagging strategies, two using the short peptide HA and one with the fluorescent protein mCherry. The short DNA sequence encoding the HA-tag was inserted at either the beginning or the end of the SNCA gene (Fig. 1*B*) using a modified ssODN to prevent rapid degradation (Renaud et al., 2016). For the fluorescent reporter line, a longer DNA sequence encoding mCherry was introduced only at the end of the SNCA gene (Fig. 1*B*) since the N-terminal domain of α-synuclein is known to interact with lipid bilayers (Bodner et al., 2009). We utilized sgRNA target sites flanking the homology arms in the donor plasmid (Extended Data Fig. 1-6*A*) to enhance homology-directed repair (HDR) as previously suggested (Zhang et al., 2017).

10.1523/ENEURO.0093-25.2025.f1-1 Figure 1-1 Sequence of sgRNAs to edit the SNCA locus. Download Figure 1-1, DOCX file.

**Figure 2.**
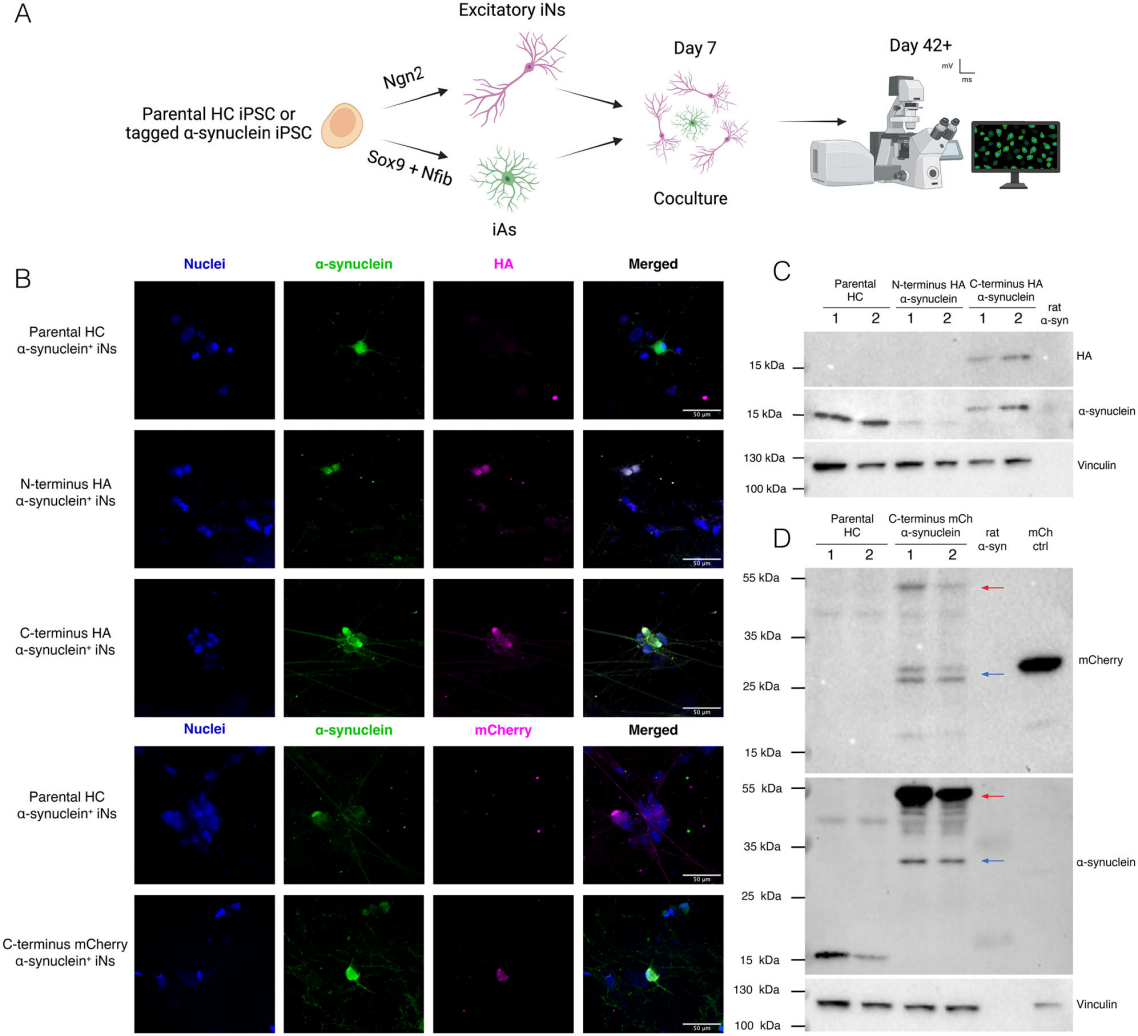
N-Terminus HA-tag affects protein expression and C-terminus mCherry-tag generates cleaved by-products. ***A***, Schematic representation of the iNs and iAs differentiation for establishing cocultures for immunocytochemistry and imaging experiments. ***B***, Representative images of mature cocultures of iNs and iAs, with iNs positive for α-synuclein and the corresponding tag, HA, or mCherry. Scale bar, 50 µm. ***C***, Western blot of protein extracts from iNs derived from the parental and HA-tagged lines, analyzed for α-synuclein and HA-tag. Protein extract from the rat brain with α-synuclein overexpressed was used as a positive control for α-synuclein antibody, and vinculin was used as loading control. ***D***, Western blot of protein extracts from iNs derived from the parental and mCherry-tagged line, analyzed for α-synuclein and mCherry. Protein extract from the rat brain with α-synuclein overexpressed and 293T cells with mCherry overexpressed were used as positive controls, and vinculin was used as the loading control. Extended Data Figure 2-1 is supporting Figure 2.

10.1523/ENEURO.0093-25.2025.f1-2 Figure 1-2 Sequence of donor DNA, with sgRNA target sequence in lower case letters, and homology arms and linker sequence separated from mCherry sequence. Download Figure 1-2, DOCX file.

**Figure 3.**
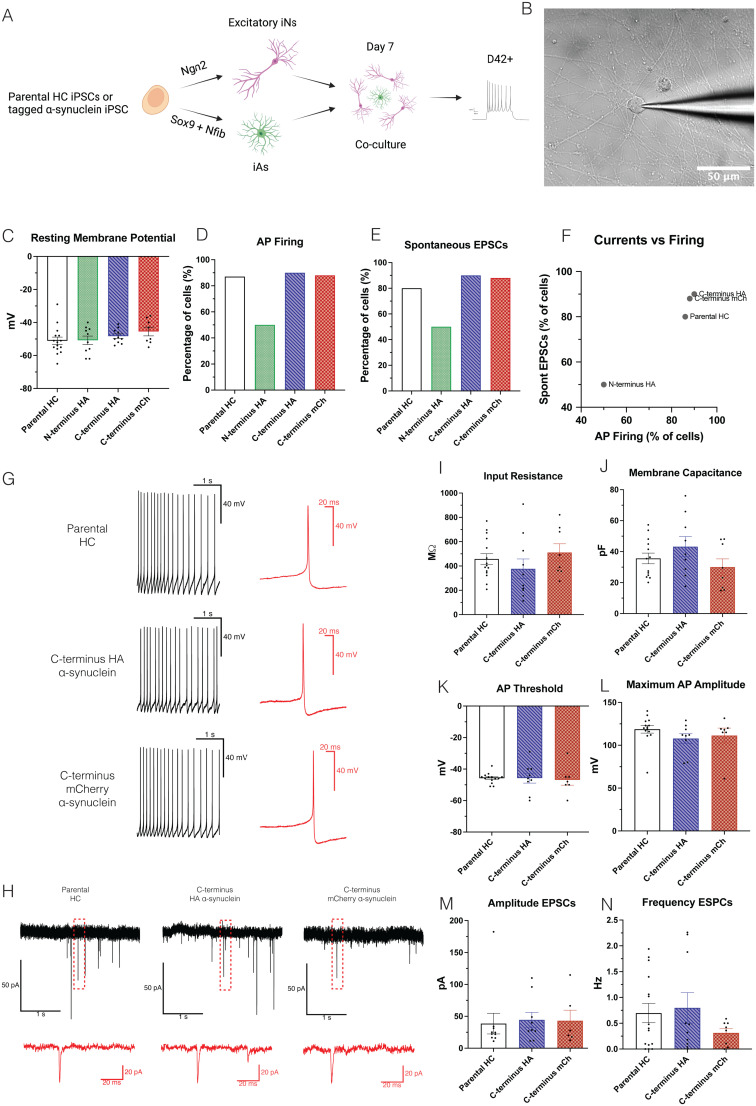
iNs derived from the C-terminus tags display normal neuronal functionality. ***A***, Schematic representation of the iNs and iAs differentiation for establishing cocultures for electrophysiological experiments. ***B***, Representative bright-field image of a patch-clamped iNs. Scale bar, 50 µm. ***C***, RMP of iNs from the different iPSC lines measured in millivolt. Bar graphs indicate mean ± SEM with *n* = 15 for the parental HC, *n* = 10 for the N-terminus HA line, *n* = 10 for the C-terminus HA line, and *n* = 8 for the C-terminus mCherry line. ***D***, The percentage of patched iNs from each iPSC line able to fire APs. ***E***, The percentage of patched iNs from each iPSC line with spontaneous excitatory postsynaptic currents. ***F***, Comparison of the percentage of patched iNs from each iPSC line able to fire APs (*X*-axis) and exhibiting spontaneous excitatory postsynaptic currents (*Y*-axis). ***G***, Representative traces of spontaneous AP firing of one iN for each iPSC line. ***H***, Representative traces of spontaneous synaptic activity of one iN for each iPSC line. ***I***, Ri of iNs from the different iPSC lines measured in megaohm. Bar graphs indicate mean ± SEM with *n* = 15 for the parental HC, *n* = 10 for C-terminus HA line, and *n* = 8 for the C-terminus mCherry line. ***J***, Cm of iNs from the different iPSC lines measured in picofarad. Bar graphs indicate mean ± SEM with *n* = 15 for the parental HC, *n* = 10 for the C-terminus HA line, and *n* = 8 for the C-terminus mCherry line. ***K***, AP threshold of iNs from the different iPSC lines measured in millivolt. Bar graphs indicate mean ± SEM with *n* = 14 for the parental HC, *n* = 9 for the C-terminus HA line, and *n* = 7 for the C-terminus mCherry line. ***L***, Maximal AP amplitude of iNs from the different iPSC lines measured in millivolt. Bar graphs indicate mean ± SEM with *n* = 14 for the parental HC, *n* = 9 for the C-terminus HA line, and *n* = 7 for the C-terminus mCherry line. ***M***, Amplitude of the excitatory postsynaptic currents of iNs from the different iPSC lines measured in picoampere. Bar graphs indicate mean ± SEM with *n* = 12 for the parental HC, *n* = 9 for the C-terminus HA line, and *n* = 7 for the C-terminus mCherry line. ***N***, Frequency of the excitatory postsynaptic currents of iNs from the different iPSC lines measured in hertz. Bar graphs indicate mean ± SEM with *n* = 15 for the parental HC, *n* = 10 for the C-terminus HA line, and *n* = 8 for the C-terminus mCherry line. For C-E and I-N, one-way ANOVA was used to compare the means of the electrophysiological parameters across the groups of iNs. Assumptions of normality and homogeneity of variances were verified prior to analysis. A significance level of *α* = 0.05 was used, and no statistical differences were detected. Extended Data Figure 3-1 is supporting Figure 3.

10.1523/ENEURO.0093-25.2025.f1-3 Figure 1-3 Antibody information. Showing name of antibody, host species, manufacturer, catalog number and dilutions used for different applications. Download Figure 1-3, DOCX file.

**Figure 4.**
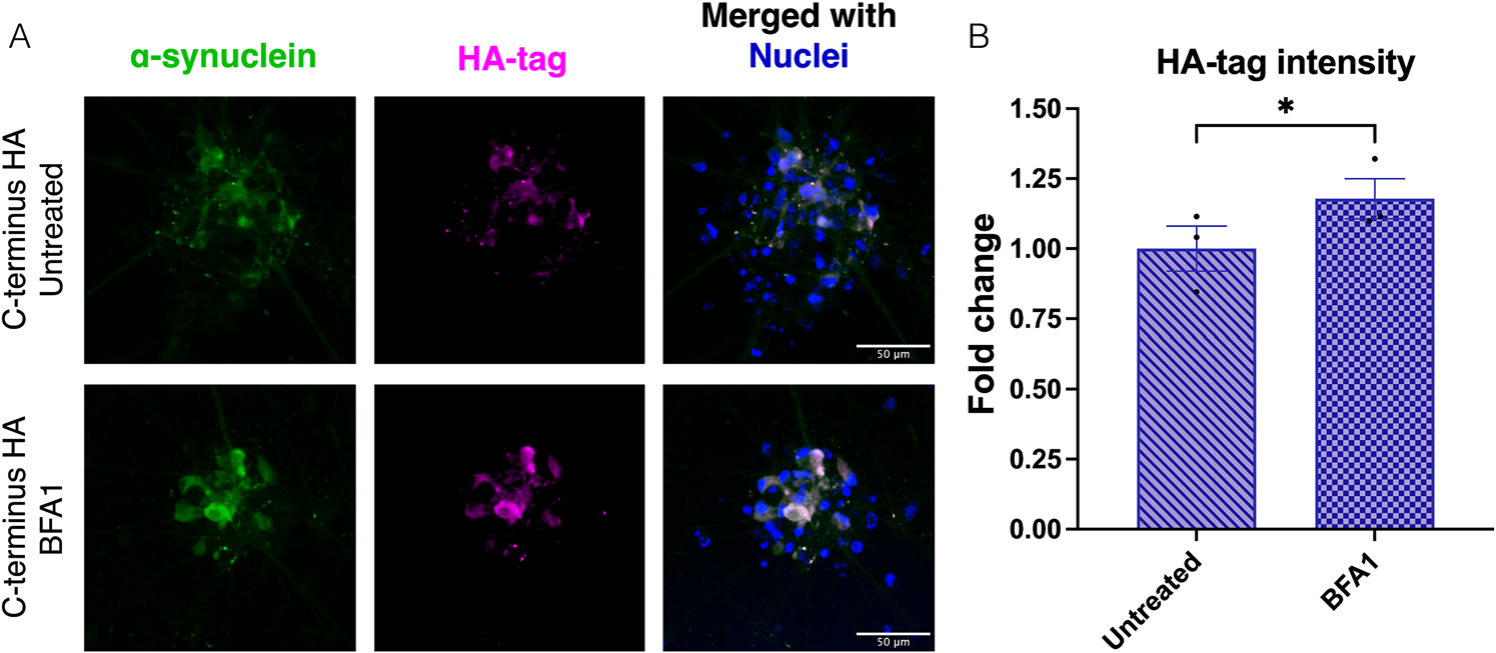
C-Terminus HA-tag iNs allow to visualize α-synuclein accumulation. ***A***, Representative immunocytochemistry images of mature cocultures of iN and iAs from the C-terminus HA-tagged iPSC line with and without BFA1 treatment. Scale bar, 50 µm. ***B***, Fold change of HA intensity in cocultures derived from the C-terminus HA-tagged iPSC line, comparing BFA1-treated to untreated cells, and normalized within each independent experiment. Bar graph indicates mean ± SEM of three independent experiments. One-tailed ratio paired *t* test was used to detect the potential increase in intensity upon treatment, with a significance level of *α* = 0.05, and a significant increase of HA-tag intensity following BFA1 treatment was detected. Extended Data Figure 4-1 is supporting Figure 4.

10.1523/ENEURO.0093-25.2025.f1-4 Figure 1-4 TaqMan probes used for real-time quantitative PCR in this study. Download Figure 1-4, DOCX file.

10.1523/ENEURO.0093-25.2025.f1-5 Figure 1-5 Generation and characterization of the parental HC iPSC line. **A**: Schematic representation of mRNA-based reprogramming of HC fibroblasts towards iPSCs. **B**: Representative G-band karyotyping analyses of the HC iPSCs. **C**: Representative immunocytochemistry images of HC iPSCs, positive for the markers of undifferentiated state TRA-1-81, NANOG, OCT3/4 and SOX2. **D**: Representative immunocytochemistry images of FOXA2 and SOX17, markers of endoderm (top panels), PAX6 and beta-III-tubulin (TUBB3), markers of ectoderm (middle panels), and Brachyury (BRA) and alpha-smooth muscle actin (α-SMA), markers of mesoderm (lower panels) for HC iPSCs differentiated towards each germ layer. Scale bar = 100 μm (C, D). Download Figure 1-5, TIF file.

10.1523/ENEURO.0093-25.2025.f1-6 Figure 1-6 Donor plasmid used for mCherry tagging and results of the off-target analysis of the tagged iPSC lines. **A**: Schematic representation of the donor plasmid used for introducing mCherry into the coding sequence of the SNCA gene, with the sgRNA115 target sequence flanking the homology arms. **B**: Chromatogram showing the correct sequence for the top-5 predicted off-target (OT) sites for sgRNA84 for HC and N-terminus HA tagged iPSC lines. **C**: Chromatogram showing the correct sequence for the top-5 predicted off-target sites for sgRNA115 for HC and the two C-terminus tagged iPSC lines. Download Figure 1-6, TIF file.

10.1523/ENEURO.0093-25.2025.f1-7 Figure 1-7 Efficiency of the sgRNAs targeting the different regions of the SNCA gene. Each sgRNA was cloned into both the pX459 (wild-type Cas9) and the pX462 (nickase Cas9) plasmids. The pX462 plasmids were always used in combinations of sgRNAs targeting the sense and antisense strands. Download Figure 1-7, DOCX file.

10.1523/ENEURO.0093-25.2025.f1-8 Figure 1-8 Summary of the number of clones examined and identified for each tag and location of integration within the SNCA gene. Download Figure 1-8, DOC file.

10.1523/ENEURO.0093-25.2025.f1-9 Figure 1-9 Trilineage differentiation of the tagged iPSC lines. **A**: Representative immunocytochemistry images of endoderm markers FOXA2 and SOX17 from the trilineage differentiation of the tagged iPSC lines. **B**: Representative immunocytochemistry images of ectoderm markers PAX6 and beta-III-tubulin (TUBB3) from the trilineage differentiation of the tagged iPSC lines. **C**: Representative immunocytochemistry images of mesoderm markers Brachyury (BRA) and alpha-smooth muscle actin (α-SMA) from the trilineage differentiation of the tagged iPSC lines. Scale bar = 100 μm (A-C). Download Figure 1-9, TIF file.

To target the N- and C-terminus, four different sgRNAs located around the start and stop codon, respectively, were generated and tested for their efficiency to generate indels in the HC iPSC line (Extended Data Fig. 1-7). Our results showed that the most efficient sgRNAs were promoting mainly small indels (Extended Data Fig. 1-7). We also tested combinations of sgRNAs together with a nickase Cas9, which has reduced off-target effects, and although efficiencies were high for some combinations, most indels were above 20 bp (Extended Data Fig. 1-7), removing part of the homology arm sequences required for HDR. Therefore, we continued our experiments using only the wild-type Cas9 version.

After optimizing the genome editing conditions, we transfected the HC iPSC line with the plasmid containing Cas9 and the sgRNA for the corresponding region of interest in the SNCA gene, together with the HA ssODN or the mCherry donor plasmid. Cells were selected and grown at clonal density to establish SNCA-tagged iPSC clones for each strategy. We detected a total of one homozygous clone with the HA-tag at the N-terminus, three homozygous clones with the HA-tag at the C-terminus, and two homozygous clones with the mCherry-tag at the C-terminus (Extended Data Fig. 1-8). One clone per line was chosen for further characterization. We confirmed correct integration of the tags using Sanger sequencing (Fig. 1*C*). Importantly, G-band karyotyping revealed no chromosomal abnormalities for any of the newly generated lines after genome editing (Fig. 1*D*). All clones displayed similar levels of the markers of undifferentiated state POU5F1, NANOG, and SOX2, at the mRNA level (Fig. 1*E*) and OCT3/4 (encoded by POU5F1), NANOG, SOX2, and TRA-1-81 at the protein level (Fig. 1*F*), compared with the parental non-tagged HC iPSC line (Extended Data Fig. 1-5*C*). Analyses of the top five predicted off-target sites confirmed no unspecific events related to the genome editing process at these sites for any line (Extended Data Fig. 1-6*B*,*C*). Furthermore, all clones showed similar capacity as the parental HC line to differentiate toward cells of the three germ layers, endoderm, ectoderm, and mesoderm (Extended Data Fig. 1-9*A*–*C*).

Altogether, we show the successful targeting of the SNCA gene in iPSCs to add either a small 9-amino acid HA-tag or a large 236-amino acid fluorescent protein tag, mCherry, in two different regions of the gene without any apparent undesired effect in properties of the iPSCs.

### Neurons derived from C-terminal tagged iPSC lines express α-synuclein fusion protein

Next, we set out to validate the expression of the tagged α-synuclein upon neuronal differentiation. We used overexpression of Ngn2 to rapidly generate excitatory iN as previously described (Zhang et al., 2013). To promote maturation of iNs, we used overexpression of Sox9 and Nfib to rapidly generate iAs as previously reported (Canals et al., 2018) and established neuron-astrocyte cocultures (Fig. 2*A*). Immunocytochemistry analysis confirmed expression of α-synuclein in neurons from all lines, as well as HA- or mCherry-tag expression only in the corresponding genome edited neurons, in all cases with similar intracellular distribution between α-synuclein and the corresponding tag (Fig. 2*B*).

10.1523/ENEURO.0093-25.2025.f2-1 Figure 2-1 Full Western blot membranes. **A**: Full size Western Blot membrane of the parental and HA-tagged lines, stained for HA-tag. Protein extract from rat brain with α-synuclein overexpressed as positive control. **B**: Full size Western Blot membrane of the parental and HA-tagged lines, stained for α-synuclein (after stripping). Protein extract from rat brain with α-synuclein overexpressed as positive control. **C**: Full size Western Blot membrane of the parental and the mCherry-tagged line, stained for mCherry. Protein extract from rat brain with α-synuclein overexpressed and 293  T cells with mCherry overexpressed as positive controls. **D**: Full size Western Blot membrane of parental and mCherry tagged lines, stained for α-synuclein (after stripping). Protein extract from rat brain with α-synuclein overexpressed and 293  T cells with mCherry overexpressed as positive controls. **E**: Full size Western Blot membrane for all the lines stained with the loading control Vinculin. Download Figure 2-1, TIF file.

To confirm the correct size of tagged proteins, we differentiated iNs for 21 d, extracted protein and performed Western Blot analysis against α-synuclein and the corresponding tags (Fig. 2*C*,*D*; Extended Data Fig. 2-1*A*–*E*). Surprisingly, protein extracts from the N-terminus HA-tagged line displayed weak expression of α-synuclein compared with the extracts from the parental HC line and no detectable signal from the antibody against HA (Fig. 2*C*; Fig. 2-1*A*,*B*,*E*). In contrast, extracts from the C-terminus HA-tagged line showed similar expression levels to those of the untagged parental HC line, which could be detected both with antibodies against α-synuclein and HA peptide and was as expected ∼1 kDa heavier than the untagged α-synuclein (Fig. 2*C*; Extended Data Fig. 4-1*A*,*B*,*E*). For the C-terminus mCherry-tagged line, a band predicted as α-synuclein tagged with mCherry could be detected with antibodies against α-synuclein or mCherry, and at a size ∼31 kDa larger than α-synuclein alone (Fig. 2*D*, red arrows; Extended Data Fig. 2-1*C*,*D*,*E*). However, two additional bands of lower molecular weight could also be detected with antibodies against α-synuclein and mCherry, or only mCherry, suggesting that the fusion protein is cleaved within cells (Fig. 2*D*, blue arrows; Extended Data Fig. 2-1*C*,*D*).

Altogether, our data demonstrate that the C-terminus tagged lines express α-synuclein protein with the expected size depending on the tag and with normal cell distribution in neurons. Importantly, the tags allow for specific detection of tagged α-synuclein. However, the presence of cleaved forms of the fusion protein in C-terminus mCherry-tagged neurons could be a source of confounding results in subsequent analyses relying on mCherry fluorescence.

### C-terminus α-synuclein tags do not affect neuronal functionality

Next, we investigated whether tagged α-synuclein affected functional properties of neurons. We cultured iNs together with iAs for 42–52 d to promote neuronal and synaptic maturation (Fig. 3*A*). Immunocytochemistry analyses showed iNs were positive for the neuronal marker MAP2 as well as α-synuclein, while iAs were positive for the astrocytic marker vimentin (Extended Data Fig. 3-1*A*).

10.1523/ENEURO.0093-25.2025.f3-1 Figure 3-1 Additional electrophysiological data from patch clamp recordings of induced neurons from the different iPSC lines. **A**: Representative immunocytochemistry images of mature cocultures of iNs and iAs, positive for α-synuclein, neuronal marker MAP2 and astrocytic marker Vimentin (VIM). **B**: Half maximum width of action potentials of iNs from the different iPSC lines measured in ms. Bar graphs show the mean ± s.e.m. with n = 14 for the parental HC, n = 19 for C-terminus HA line, and n = 7 for C-terminus mCherry line. Scale bar = 50 μm. **C**: Maximum frequency of action potential firing of iNs from the different iPSC lines measured in Hz. Bar graphs show the mean ± s.e.m. with n = 15 for the parental HC, n = 10 for C-terminus HA line, and n = 8 for C-terminus mCherry line. **D**: Distribution of cells by action potential firing frequencies in the healthy control line. E: Distribution of cells by action potential firing frequencies in the C-terminus HA line. F: Distribution of cells by action potential firing frequencies in the C-terminus mCherry line. For B and C, one-way ANOVA was used to compare the means of the electrophysiological parameters across the groups of neurons. Assumptions of normality and homogeneity of variances were verified prior to analysis. A significance level of α = 0.05 was used and no statistical differences were detected. Download Figure 3-1, TIF file.

To analyze functional properties of iNs derived from the parental HC and the three α-synuclein tagged lines, we performed whole-cell patch–clamp recordings (Fig. 3*B*) to assess passive and active electrophysiological properties of iNs at 42–52 d of differentiation. The RMP for the parental HC line was −51.07 ± 2.22 mV (Fig. 3*C*), which is comparable with that found in the literature for iNs in coculture with iAs (Canals et al., 2023). RMP for the tagged lines was −50.80 ± 2.56 mV for the N-terminus HA line, −48.30 ± 1.38 mV for the C-terminus HA line, and −45.50 ± 2.61 mV for C-terminus mCherry, with no significant differences when compared with the parental HC line (Fig. 3*C*). These findings suggest functional maturity of neurons derived from all lines and that their passive electrical properties were preserved.

We next examined spontaneous APs and spontaneous excitatory postsynapatic currents (EPSCs) from iNs of all lines (Fig. 3*D*–*H*). We found that for iNs derived from the parental HC line, 87 and 80% of the cells displayed APs and EPSCs, respectively (Fig. 3*D*–*F*). Ninety percent of iNs derived from the C-terminus HA-tagged line displayed both APs and EPSCs, while 88% of iNs differentiated from the C-terminus mCherry-tagged line exhibited both APs and EPSCs (Fig. 3*D*–*F*), indicating comparable excitability and synaptic function to that of the iNs obtained from the parental HC line (Fig. 3*F*). However, only 50% of iNs derived from the N-terminus HA-tagged line had APs and EPSCs, indicating diminished synaptic activity (Fig. 3*F*). The impaired synaptic activity of the N-terminus HA-tagged neurons suggests that the N-terminus tag could affect α-synuclein function at the synapse and the line was therefore discarded from further electrophysiological analysis.

The passive properties, Ri (Fig. 3*I*) and Cm (Fig. 3*J*), of the iNs derived from the parental HC line were similar to that of previous reports using the same coculture system (Canals et al., 2023). In addition, the two C-terminus tagged lines displayed similar values to the parental HC line without any significant differences (Fig. 3*I*,*J*), indicating preserved electrical characteristics during differentiation in the tagged lines. Similarly, analysis of the AP properties, including AP threshold (Fig. 3*K*), maximum AP amplitudes (Fig. 3*L*), half-maximal width (Extended Data Fig. 3-1*B*), the maximum frequency of firing (Extended Data Fig. 3-1*C*), and the distribution of AP firing frequencies per cell (Extended Data Fig. 3-1*D*–*F*), showed that iNs derived from the tagged lines did not present any significant difference compared with iNs from the parental HC line, suggesting that AP characteristics are not affected by the tags. Moreover, analysis of spontaneous EPSC amplitudes (Fig. 3*M*) and EPSC frequency (Fig. 3*N*) indicated that spontaneous synaptic current characteristics of iNs differentiated from the parental HC lines were similar to previous reports (Zhang et al., 2013) and the C-terminus tagged lines were comparable with the parental HC line, therefore displaying similar connectivity.

Thus, our data clearly show that the C-terminus HA- and mCherry-tags in α-synuclein did not affect functional properties of iPSC-derived neurons. They also suggest that the N-terminus HA-tag impacts neuronal function, indicating it may not be a good candidate site for modification in α-synuclein

### *SNCA*-tagged iPSC lines can be used to detect α-synuclein aggregation in neurons upon lysosomal dysfunction

After having established the C-terminus HA-tag as a feasible option, considering that the N-terminus HA-tag affects neuronal function while the mCherry-tag generates cleaved protein products, we aimed at providing proof of principle of how this iPSC line can be used to model synucleinopathies and to visualize α-synuclein distribution and accumulation. For that purpose, we treated cocultures of iNs and iAs with BFA1, to inhibit autophagy (mimicking a major lysosomal impairment), or CBE to inhibit GCase activity (mimicking Gaucher disease), both promoting lysosomal dysfunction and subsequent α-synuclein accumulation (Cleeter et al., 2013). Immunocytochemistry analyses of HA-tag and α-synuclein followed by confocal microscopy imaging showed a clear signal in the C-terminus HA-tagged iNs with very similar distribution to α-synuclein and without positive signal in iNs derived from the untagged HC line (Fig. 4*A*; Extended Data Fig. 4-1*A*). We performed quantifications of HA intensity comparing BFA1- or CBE-treated and untreated cocultures generated with the C-terminus HA-tag lines (Fig. 4*B*; Extended Data Fig. 4-1*B*). While no significant difference could be observed following the CBE treatment (Extended Data Fig. 4-1*B*), upon treatment with BFA1, a significant increase in HA intensity could be detected (Fig. 4*B*), suggesting that HA-tagged α-synuclein can be used to visualize accumulation in neurons.

10.1523/ENEURO.0093-25.2025.f4-1 Figure 4-1 CBE treatment of cocultures from C-terminus HA-tagged line **A**: Representative immunocytochemistry images of mature cocultures of iN and iAs from the C-terminus HA-tagged iPSC line following treatment with CBE. Scale bar = 50 μm. **B**: Fold change of HA intensity in cocultures derived from the C-terminus HA-tagged iPSC line, comparing CBE treated to untreated cells (from Fig 4), and normalized within each experiment. Bar graph shows mean ± s.e.m. of 3 independent experiments. One-tailed ratio paired t-test was used to analyze the potential increase in intensity upon treatment, with a significance level of α = 0.05, and no significant difference was detected. Download Figure 4-1, TIF file.

In summary, the C-terminus HA-tagged α-synuclein line can be used to detect α-synuclein using the tag with high specificity and can be a useful tool to visualize accumulation upon lysosomal dysfunction without overexpression of α-synuclein.

## Discussion

Here we developed different α-synuclein iPSC reporter lines. After quality and functional assessment, we identified a C-terminus HA-tag as the best option for not interfering with α-synuclein protein expression and neuronal function. Moreover, we have shown that this reporter line can be used to monitor α-synuclein intracellularly and detect the protein after promoting accumulation.

Previous efforts to generate tagged α-synuclein reporters include transfection approaches to overexpress GFP-tagged α-synuclein in neuroblastoma cells (Hansen et al., 2011) and a mouse model with GFP-tagged endogenous α-synuclein (Hansen et al., 2013), both developed by the same lab. However, it is important to consider that GFP is not resistant to the acidic pH of the lysosome (Rodriguez et al., 2017), thereby hampering its use to study aggregation within lysosomes. In contrast, mCherry has been shown to be more stable at low pH (Doherty et al., 2010). Despite the choice to use the more stable mCherry instead of GFP, our findings indicated that the α-synuclein–mCherry fusion protein was cleaved intracellularly, generating different by-products. Supporting this observation, previous work showed that other mCherry-tagged proteins could be cleaved, generating by-products that can be fluorescent and only partially retain the functions of the native protein (Huang et al., 2014). Fluorescence from such by-products could lead to confounding results when using α-synuclein–mCherry or similar fluorescent protein tagged lines for visualizing α-synuclein expression, localization, and aggregation. Although we did not observe any difference in electrophysiological properties of neurons derived from the α-synuclein–mCherry line, we cannot rule out a loss of normal function of α-synuclein upon tagging with mCherry. Taken together, our data strongly indicate that fluorescent proteins are not optimal tags for α-synuclein.

Alternative approaches to visualize α-synuclein have relied on overexpression of the protein tagged with either HA, V5 (Klucken et al., 2006), or Myc (Desplats et al., 2009). Nevertheless, these approaches do not allow for specific visualization of the endogenous protein, and strategies using overexpression of tagged α-synuclein have been shown to be toxic (Kamp et al., 2010) and affect synaptic function (White et al., 2024).

Given the known interaction between the amphipathic N-terminal region of α-synuclein and lipid bilayers (Braun et al., 2017) and the size of the mCherry-tag (236 amino acids), we decided against trying to introduce mCherry at the N-terminus. On the other hand, as the HA epitope only consists of nine amino acids, we hypothesized that it could be small enough to not affect the properties of this domain. However, our data from Western blotting and immunofluorescence experiments suggested that the HA-tag has a negative effect on expression of α-synuclein, with the amount of total protein being substantially reduced. Moreover, electrophysiological analysis further indicated that the N-terminus tagging impaired functional properties of neurons, suggesting an importance of the amphipathic N-terminal region of α-synuclein for regulation of neuronal function. Altogether, our data support that the N-terminal domain of α-synuclein is not a good target for introducing a reporter, regardless of the size of the tag.

In contrast to the C-terminus mCherry and N-terminus HA-tagged lines, C-terminus HA- tagging allowed expression and visualization of α-synuclein and did not affect electrophysiological properties of neurons. Therefore, we suggest that a small tag, such as HA, at the C-terminus of α-synuclein should be prioritized over other approaches when generating α-synuclein reporters. The only, to our knowledge, other α-synuclein iPSC reporter line previously generated, indeed, used a similar strategy, with a FLAG peptide at the C-terminus (di Domenico et al., 2019). However, whether the tag affected properties of the protein or the functionality of neurons was not investigated. Together, these studies support the use of short peptides to tag α-synuclein C-terminus.

Fluorescent reporters allow live cell imaging, while with other tags this is not as straightforward, which is a limitation of HA tagging. Nevertheless, progress made with antibody-derived probes, such as the HA frankenbody (Zhao et al., 2019), could allow live cell imaging also of HA-tagged proteins. Subsequent studies should determine the feasibility of live cell imaging of HA-tagged α-synuclein using this approach.

One of the potential uses of α-synuclein tagged iPSC lines is their use in studying synucleinopathies and other diseases such as lysosomal storage diseases, which can present secondary lysosomal aggregation of α-synuclein (Mazzulli et al., 2011; Winder-Rhodes et al., 2012). We employed BFA1 to inhibit autophagosome–lysosome fusion (Mauvezin and Neufeld, 2015) and CBE to mimic the effect of GBA1 mutations, which causes Gaucher’s disease and predisposition to Parkinson's disease. BFA1 treatment can be considered the harsher of the two, severely inhibiting autophagic function (Mauvezin and Neufeld, 2015). Following 48 h of treatment, we could detect a significant increase in HA intensity in treated cells compared with untreated controls, confirming the ability of the C-terminal HA line to be used for detecting endogenous α-synuclein accumulation in neurons. CBE treatment, which impairs the enzymatic activity of GCase, mimicking the lysosomal defect seen in neuropathic Gaucher's disease (Cleeter et al., 2013; Vardi et al., 2016), did not significantly change HA intensity following 21 d of treatment. Given that α-synuclein is a secondary aggregate that occurs downstream of initial lysosomal impairment caused by the CBE treatment, it is possible that longer time is required to see a significant effect of this intervention on HA intensity. With these experiments, we demonstrate the utility of the HA-tagged line we generated.

Importantly, C-terminus HA-tagged α-synuclein will allow investigating α-synuclein aggregation under physiological expression levels (rather than overexpression) in human in vitro models, preventing experimental artifacts related to the lack of specificity of antibodies in previous studies (Leupold et al., 2022). Another alternative to standard antibodies is the use of a reporter nanobody (Gerdes et al., 2020), although its application to visualize endogenous α-synuclein in human neurons remains to be proven. Thus, the C-terminus HA-tagged iPSC line we generated could be used with pharmacological interventions, as shown here, or in combination with CRISPR/Cas9 to introduce disease-causing mutations in the SNCA gene or lysosomal enzymes to study α-synuclein aggregation under physiological protein levels.

In conclusion, we have identified an optimal strategy and method of HA tagging the C-terminus domain of α-synuclein in iPSCs without affecting protein properties or neuronal functionality. This method can easily be combined with iPSC differentiation toward brain cells to provide a better understanding of the roles of α-synuclein in health and in diseases such as Parkinson's disease, dementia with Lewy bodies, or neuronopathic lysosomal storage disorders.

## Synthesis

Reviewing Editor: Jennifer Dulin, Texas A&M University Decisions are customarily a result of the Reviewing Editor and the peer reviewers coming together and discussing their recommendations until a consensus is reached. When revisions are invited, a fact-based synthesis statement explaining their decision and outlining what is needed to prepare a revision will be listed below. The following reviewer(s) agreed to reveal their identity: NONE.

This manuscript describes the generation of a new reporter line for alpha-synuclein. Unfortunately, some of the the images are of too poor resolution to draw conclusions about the data, which has limited the ability of the reviewers to assess the manuscript. Fluorescent images are of very low resolution and text in other figures is unreadable. The manuscript should be resubmitted with higher-resolution figures to undergo additional review. Additionally, while it may be helpful for others to know the HA tag seems to work well, a proof of concept experiment in a relevant question in synucleinopathies would significantly improve the value of having the line.

Comments:

This is a solid work to generate a iPSC bassed alpha-synuclein reporter line. Pluripotency, differentiation potential and neuronal function are carefully checked. The important take home message is that the HA tag is superior to the fluorescence methods used. The images provided for alpha-synuclein are provided in very low resolution, to difficult to judge the value here. The experiments provided with the lines include differentiation in neurons and astrocytes using LV based systems and alpha-synuclein analysis.

## Author Response

Dear Dr. Dulin, Thank you very much for considering our manuscript entitled "Generation of iPSC lines with tagged α-synuclein to visualize the endogenous protein in human cellular models of neurodegenerative disorders" and for the reviewers' constructive assessment. We are very happy that our manuscript is considered very interesting, useful for the field as well as for studies of neurological disorders.

We have now addressed all issues brought up by the reviewers, including high-quality images for all the figures and a quantification in the proof-of-concept experiment promoting accumulation of α-synuclein after pharmacological interventions. We hope that with these new experiments, data and changes in the text, our manuscript will now be considered suitable for publication in eNeuro.

Please find below a point-by-point description, in red, of how we have addressed the comments from the reviewers. Changes in the manuscript are also indicated in red.

Yours Sincerely.

Reviewers' comments:

This manuscript describes the generation of a new reporter line for alpha-synuclein.

Unfortunately, some of the images are of too poor resolution to draw conclusions about the data, which has limited the ability of the reviewers to assess the manuscript. Fluorescent images are of very low resolution and text in other figures is unreadable. The manuscript should be resubmitted with higher-resolution figures to undergo additional review. Additionally, while it may be helpful for others to know the HA tag seems to work well, a proof-of-concept experiment in a relevant question in synucleinopathies would significantly improve the value of having the line.

This is a solid work to generate a iPSC based alpha-synuclein reporter line. Pluripotency, differentiation potential and neuronal function are carefully checked. The important take home message is that the HA tag is superior to the fluorescence methods used. The images provided for alpha-synuclein are provided in very low resolution, too difficult to judge the value here.

The experiments provided with the lines include differentiation in neurons and astrocytes using LV based systems and alpha-synuclein analysis.

We thank the reviewers for their positive view on the work presented in this manuscript. We fully agree with the reviewers on the poor quality of some of the images. We provided good quality images, but there was an issue arising from the automatic compiling of all documents to generate the version of the manuscript for revision. Nevertheless, we have now generated the highest quality images possible for all the figures in the manuscript to improve the presentation of the data.

We completely agree with the reviewer that a proof-of-concept experiment relevant for synucleinopathies would be useful. We originally presented pharmacological interventions to promote accumulation of α-synuclein by either using CBE to mimic Gaucher disease, a lysosomal storage disorder with secondary α-synuclein accumulation, or Bafilomycin A1, an autophagy blocker, to promote an impairment in the recycling system of the cell and promote protein accumulation, including α-synuclein. In the revised version, we clarify this proof-ofconcept experiment in the text, provide quantification of changes in HA intensity upon treatment and show a significant increase in HA intensity with Bafilomycin A1 treatment, while CBE had no significant effect. We hypothesize that Bafilomycin A1 blocking autophagy represents a more severe intervention than CBE inhibiting one unique lysosomal enzyme, and thus we see stronger effect with BFA1. We firmly believe that these experiments show the relevance for synucleinopathies and provide a proof-of-concept for how this line and concept will be useful for the field.

Relevant changes in the methods, results and discussion sections have been added according to this quantification and are highlighted in red in the text. Moreover, Fig 4 has been divided in Fig 4 and Fig 4-1.
