# Supplementary material for: Generation of iPSC Lines with Tagged α-Synuclein for Visualization of Endogenous Protein in Human Cellular Models of Neurodegenerative Disorders
Source: eNeuro. 2025 Jun 10;12(6):ENEURO.0093-25.2025. doi: 10.1523/ENEURO.0093-25.2025 (PMC12186606; doi:10.1523/ENEURO.0093-25.2025)
Supplement: Figure 1-1 — Sequence of sgRNAs to edit the SNCA locus. Download Figure 1-1, DOCX file. [file eneuro-12-ENEURO.0093-25.2025-s003.docx]

Figure 1-1: Sequence of sgRNAs to edit the *SNCA* locus

|  | **Name** | **sgRNA sequence** | **PAM sequence** |
| --- | --- | --- | --- |
| **N-terminus** | sgRNA175 | 5’-GCTGCTGAGAAAACCAAACA-3’ | GGG |
|  | sgRNA141 | 5’-AGGACTTTCAAAGGCCAAGG-3’ | AGG |
|  | sgRNA111 | 5’-TCCTTTCATGAATACATCCA-3’ | TGG |
|  | sgRNA84 | 5’-TGAATTCCTTTACACCACAC-3’ | TGG |
| **C-terminus** | sgRNA115 | 5’-TGGGAGCAAAGATATTTCTT-3’ | AGG |
|  | sgRNA108 | 5’-AAAGATATTTCTTAGGCTTC-3’ | AGG |
|  | sgRNA81 | 5’-GATTTTTCTAATATTAGGAA-3’ | GGG |
|  | sgRNA76 | 5’-TTTTTGATTTTTCTAATATT-3’ | AGG |
