# Supplementary material for: Generation of iPSC Lines with Tagged α-Synuclein for Visualization of Endogenous Protein in Human Cellular Models of Neurodegenerative Disorders
Source: eNeuro. 2025 Jun 10;12(6):ENEURO.0093-25.2025. doi: 10.1523/ENEURO.0093-25.2025 (PMC12186606; doi:10.1523/ENEURO.0093-25.2025)
Supplement: Figure 1-2 — Sequence of donor DNA, with sgRNA target sequence in lower case letters, and homology arms and linker sequence separated from mCherry sequence. Download Figure 1-2, DOCX file. [file eneuro-12-ENEURO.0093-25.2025-s004.docx]

Figure 1-2: Sequence of donor DNA, with sgRNA target sequence in lower case letters, and homology arms and linker sequence separated from mCherry sequence.

| **Donor DNA** |  | **Sequence** |
| --- | --- | --- |
| *HA tag* |  | 5’-TACCCATACGATGTTCCAGATTACGCT-3’ |
| *mCherry tag* | HAL | 5’-aagaaatatctttgctcccaAAGCCATAGATGAAGACGAAATTTTCAACTGGGAGAGTGAAAGTAGGGAAAATGTATCTTGCCTTCAAACATCTTAATTTCCTTCTGAGAATTAGAGCATCTTAGTCTGGAAAAGGCTTTATAGACAGCTTGATTTTGTTCTCACATTTTACAGGTGAAGAAACTGAGAACCAGACAGTCCAACTTATTTGTCCTACCAAACTAGGTATATGATCATTAAATGGTGCATCCGGATCAGAACCTAGATATTTTAACTCTGACTACTACTGTAATTCACTTTTATATCAGACAAGAAAGACACAACTATTAAAAATAAGATAATATTTGCTGCAGAATATTTGCAAAAACATTGATTGTAAATTTTAGTGTAAGTGGGGAGCCATTTCCTATCTCATTGGCTGTCAGTGCTGATGCGTAATTGAAACTTATACTAACAGTGTGTGCTGTCTTTTTGATTTTTCTAATATTAGGAAGGGTATCAAGACTACGAACCTGAAGCC |
|  | Linker | GCGCCTGCACCCGCCCCAGCTCCGGCGCCTGCACCCGCCCCA |
|  | CDS | ATGGTGAGCAAGGGCGAGGAGGATAACATGGCCATCATCAAGGAGTTCATGCGCTTCAAGGTGCACATGGAGGGCTCCGTGAACGGCCACGAGTTCGAGATCGAGGGCGAGGGCGAGGGCCGCCCCTACGAGGGCACCCAGACCGCCAAGCTGAAGGTGACCAAGGGTGGCCCCCTGCCCTTCGCCTGGGACATCCTGTCCCCTCAGTTCATGTACGGCTCCAAGGCCTACGTGAAGCACCCCGCCGACATCCCCGACTACTTGAAGCTGTCCTTCCCCGAGGGCTTCAAGTGGGAGCGCGTGATGAACTTCGAGGACGGCGGCGTGGTGACCGTGACCCAGGACTCCTCCCTGCAGGACGGCGAGTTCATCTACAAGGTGAAGCTGCGCGGCACCAACTTCCCCTCCGACGGCCCCGTAATGCAGAAGAAGACCATGGGCTGGGAGGCCTCCTCCGAGCGGATGTACCCCGAGGACGGCGCCCTGAAGGGCGAGATCAAGCAGAGGCTGAAGCTGAAGGACGGCGGCCACTACGACGCTGAGGTCAAGACCACCTACAAGGCCAAGAAGCCCGTGCAGCTGCCCGGCGCCTACAACGTCAACATCAAGTTGGACATCACCTCCCACAACGAGGACTACACCATCGTGGAACAGTACGAACGCGCCGAGGGCCGCCACTCCACCGGCGGCATGGACGAGCTGTACAAGTAG |
|  | HAR | GAAATATCTTTGCTCCCAGTTTCTTGAGATCTGCTGACAGATGTTCCATCCTGTACAAGTGCTCAGTTCCAATGTGCCCAGTCATGACATTTCTCAAAGTTTTTACAGTGTATCTCGAAGTCTTCCATCAGCAGTGATTGAAGTATCTGTACCTGCCCCCACTCAGCATTTCGGTGCTTCCCTTTCACTGAAGTGAATACATGGTAGCAGGGTCTTTGTGTGCTGTGGATTTTGTGGCTTCAATCTACGATGTTAAAACAAATTAAAAACACCTAAGTGACTACCACTTATTTCTAAATCCTCACTATTTTTTTGTTGCTGTTGTTCAGAAGTTGTTAGTGATTTGCTATCATATATTATAAGATTTTTAGGTGTCTTTTAATGATACTGTCTAAGAATAATGACGTATTGTGAAATTTGTTAATATATATAATACTTAAAAATATGTGAGCATGAAACTATGCACCTATAAATACTAAATATGAAATTTTACCATTTTGtgggagcaaagatatttctt  -3’ |

HAL = left homology arm; CDS = coding sequence; HAR = right homology arm
