# Supplementary material for: Generation of iPSC Lines with Tagged α-Synuclein for Visualization of Endogenous Protein in Human Cellular Models of Neurodegenerative Disorders
Source: eNeuro. 2025 Jun 10;12(6):ENEURO.0093-25.2025. doi: 10.1523/ENEURO.0093-25.2025 (PMC12186606; doi:10.1523/ENEURO.0093-25.2025)
Supplement: Figure 1-3 — Antibody information. Showing name of antibody, host species, manufacturer, catalog number and dilutions used for different applications. Download Figure 1-3, DOCX file. [file eneuro-12-ENEURO.0093-25.2025-s005.docx]

Figure 1-3: Antibody information. Showing name of antibody, host species, manufacturer, catalog number and dilutions used for different applications.

| **Antibody** | **Host Species** | **Manufacturer** | **Catalog number** | **Dilution** |
| --- | --- | --- | --- | --- |
| TRA-1-1 | Mouse | Millipore | MAB4381 | 1:200 ICC |
| NANOG | Rabbit | Abcam | ab21624 | 1:100 ICC |
| OCT 3/4 (C10) | Mouse | Santa Cruz Biotechnology | sc-5279 | 1:100 ICC |
| SOX2 | Goat | R&D Systems | AF2018 | 1:100 ICC |
| SOX17 | Goat | R&D Systems | AF1924 | 1:100 ICC |
| hHNF-3b / FOXA2 | Goat | R&D Systems | AF2400 | 1:100 ICC |
| Brachyury | Goat | R&D Systems | AF2085 | 1:100 ICC |
| Alpha-Smooth Muscle Actin | Mouse | Sigma-Aldrich | A5228 | 1:500 ICC |
| PAX6 | Rabbit | Biolegend | 901301 | 1:300 ICC |
| Beta III Tubulin | Mouse | Merck | T8660 | 1:500 ICC |
| MAP2 | Chicken | Abcam | ab5392 | 1:5000 ICC |
| Vimentin (V9) | Mouse | Dako | M0725 | 1:500 ICC |
| α-synuclein (MJFR1) | Rabbit | Abcam | ab138501 | 1:1000 ICC 1:2000 WB |
| HA-epitope (2-2-14) | Mouse | Invitrogen | 26183 | 1:500 ICC  1:1000 WB |
| mCherry (1C51) | Mouse | Abcam | ab125096 | 1:500 ICC  1:2000 WB |
| Cathepsin D | Goat | R&D Systems | af1014 | 1:100 ICC |
| Vinculin (EPR8185) | Rabbit | Abcam | ab129002 | 1:20000 WB |
| Alexa fluor 488 anti-Mouse (IgM) | Donkey | Jackson Immunoreseach | 715-545-140 | 1:500 |
| Alexa Fluor 568 anti-Rabbit | Donkey | Thermofisher Scientific | A-10042 | 1:500 |
| Alexa Fluor 488 anti-Mouse (IgG) | Donkey | Thermofisher Scientific | A-21202 | 1:500 |
| Alexa Fluor 568 anti-Goat | Donkey | Thermofisher Scientific | A-11057 | 1:500 |
| Alexa Fluor 488 anti-rabbit | Donkey | Thermofisher Scientific | A-21206 | 1:500 |
| Alexa Fluor 568 anti-Mouse | Donkey | Thermofisher Scientific | A-10037 | 1:500 |
| Alexa Fluor 647 Anti-Chicken | Donkey | Jackson Immunoreseach | 703-605-155 | 1:500 |
| Alexa Fluor 488  anti-Goat | Donkey | Thermofisher Scientific | A-21202 | 1:500 |
| Alexa Fluor 647 anti-Rabbit | Donkey | Thermofisher Scientific | A31573 | 1:500 |
| anti-rabbit HRP | Mouse | Santa Cruz Biotechnology | sc-2357 | 1:5000 |
| anti-mouse HRP | Goat | Santa Cruz Biotechnology | sc-516102 | 1:5000 |

ICC = immunocytochemistry; WB = Western Blot
