# Supplementary material for: Generation of iPSC Lines with Tagged α-Synuclein for Visualization of Endogenous Protein in Human Cellular Models of Neurodegenerative Disorders
Source: eNeuro. 2025 Jun 10;12(6):ENEURO.0093-25.2025. doi: 10.1523/ENEURO.0093-25.2025 (PMC12186606; doi:10.1523/ENEURO.0093-25.2025)
Supplement: Figure 1-4 — TaqMan probes used for real-time quantitative PCR in this study. Download Figure 1-4, DOCX file. [file eneuro-12-ENEURO.0093-25.2025-s006.docx]

Figure 1-4: TaqMan probes used for real-time quantitative PCR in this study.

| **TaqMan Probe** | **Supplier** | **Catalog number** |
| --- | --- | --- |
| GADPH | Thermo Fisher Scientific | hs02786624_g1 |
| SOX2 | Thermo Fisher Scientific | hs01053049_s1 |
| NANOG | Thermo Fisher Scientific | hs02387400_g1 |
| POU5F1 | Thermo Fisher Scientific | hs00742896_s1 |
