# Supplementary figures and images for: Generation of iPSC Lines with Tagged α-Synuclein for Visualization of Endogenous Protein in Human Cellular Models of Neurodegenerative Disorders
Source: eNeuro. 2025 Jun 10;12(6):ENEURO.0093-25.2025. doi: 10.1523/ENEURO.0093-25.2025 (PMC12186606; doi:10.1523/ENEURO.0093-25.2025)

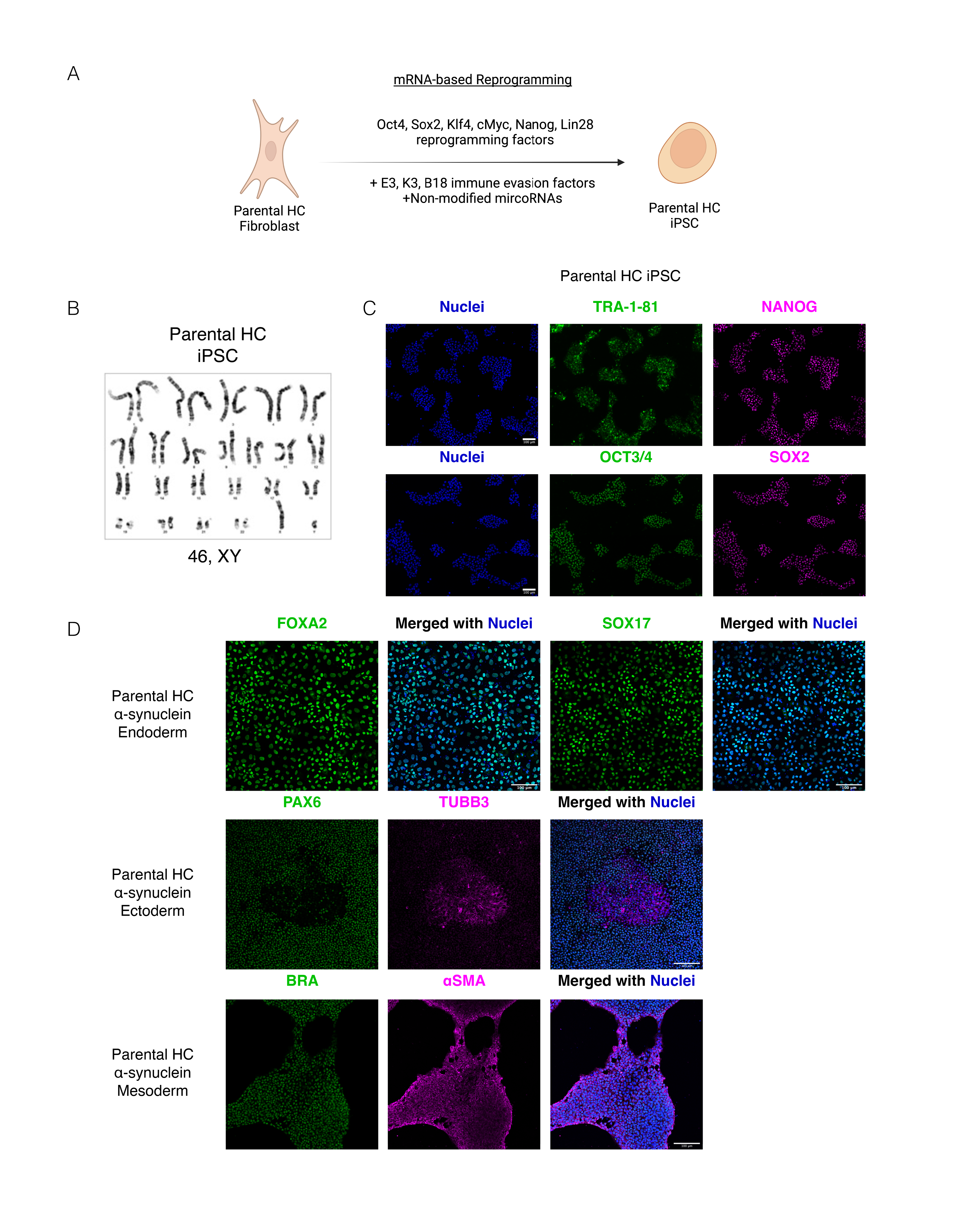

Supplement: Figure 1-5 — Generation and characterization of the parental HC iPSC line. A: Schematic representation of mRNA-based reprogramming of HC fibroblasts towards iPSCs. B: Representative G-band karyotyping analyses of the HC iPSCs. C: Representative immunocytochemistry images of HC iPSCs, positive for the markers of undifferentiated state TRA-1-81, NANOG, OCT3/4 and SOX2. D: Representative immunocytochemistry images of FOXA2 and SOX17, markers of endoderm (top panels), PAX6 and beta-III-tubulin (TUBB3), markers of ectoderm (middle panels), and Brachyury (BRA) and alpha-smooth muscle actin (α-SMA), markers of mesoderm (lower panels) for HC iPSCs differentiated towards each germ layer. Scale bar = 100 μm (C, D). Download Figure 1-5, TIF file. [file eneuro-12-ENEURO.0093-25.2025-s007.tif]

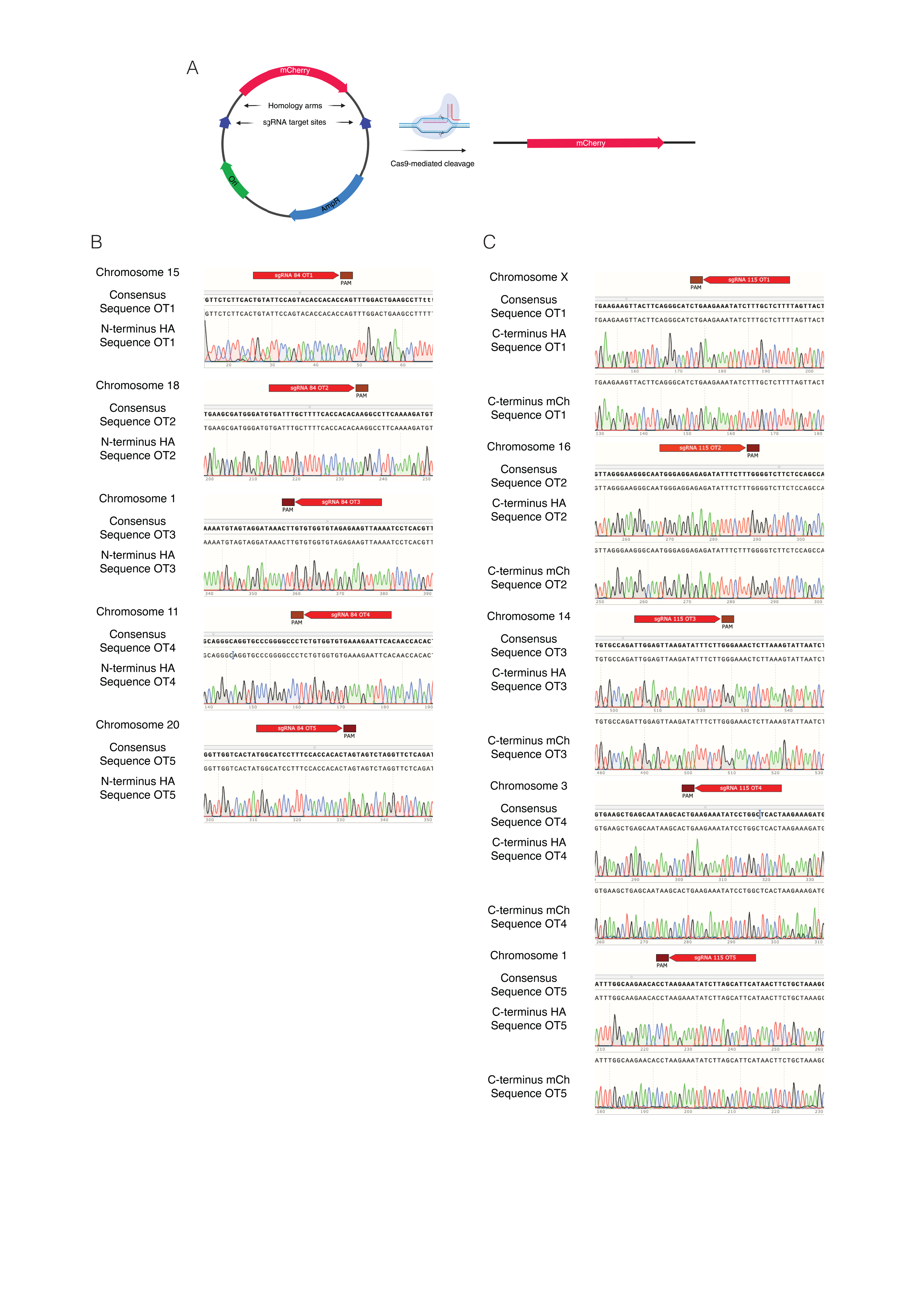

Supplement: Figure 1-6 — Donor plasmid used for mCherry tagging and results of the off-target analysis of the tagged iPSC lines. A: Schematic representation of the donor plasmid used for introducing mCherry into the coding sequence of the SNCA gene, with the sgRNA115 target sequence flanking the homology arms. B: Chromatogram showing the correct sequence for the top-5 predicted off-target (OT) sites for sgRNA84 for HC and N-terminus HA tagged iPSC lines. C: Chromatogram showing the correct sequence for the top-5 predicted off-target sites for sgRNA115 for HC and the two C-terminus tagged iPSC lines. Download Figure 1-6, TIF file. [file eneuro-12-ENEURO.0093-25.2025-s008.tif]

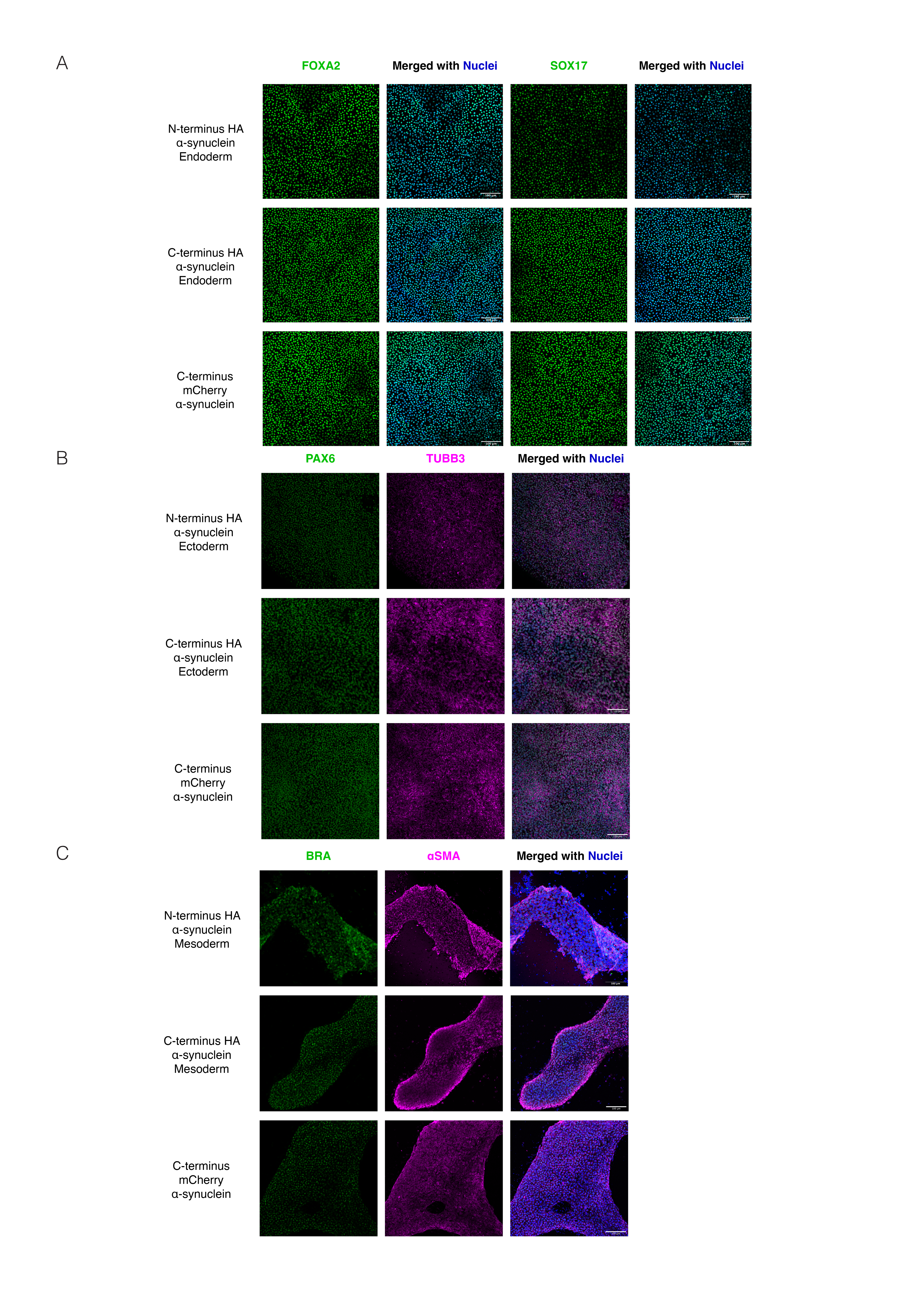

Supplement: Figure 1-9 — Trilineage differentiation of the tagged iPSC lines. A: Representative immunocytochemistry images of endoderm markers FOXA2 and SOX17 from the trilineage differentiation of the tagged iPSC lines. B: Representative immunocytochemistry images of ectoderm markers PAX6 and beta-III-tubulin (TUBB3) from the trilineage differentiation of the tagged iPSC lines. C: Representative immunocytochemistry images of mesoderm markers Brachyury (BRA) and alpha-smooth muscle actin (α-SMA) from the trilineage differentiation of the tagged iPSC lines. Scale bar = 100 μm (A-C). Download Figure 1-9, TIF file. [file eneuro-12-ENEURO.0093-25.2025-s011.tif]

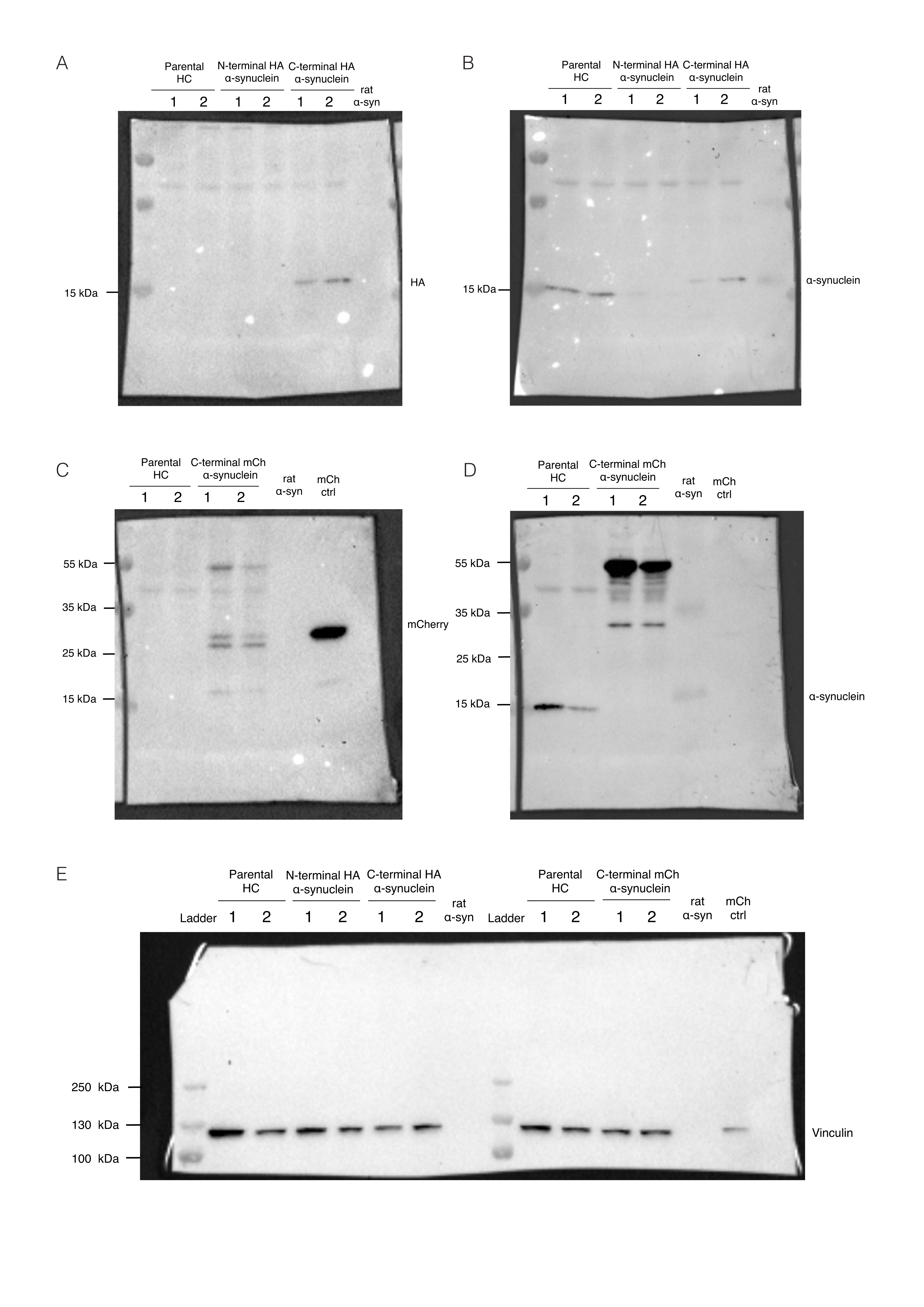

Supplement: Figure 2-1 — Full Western blot membranes. A: Full size Western Blot membrane of the parental and HA-tagged lines, stained for HA-tag. Protein extract from rat brain with α-synuclein overexpressed as positive control. B: Full size Western Blot membrane of the parental and HA-tagged lines, stained for α-synuclein (after stripping). Protein extract from rat brain with α-synuclein overexpressed as positive control. C: Full size Western Blot membrane of the parental and the mCherry-tagged line, stained for mCherry. Protein extract from rat brain with α-synuclein overexpressed and 293 T cells with mCherry overexpressed as positive controls. D: Full size Western Blot membrane of parental and mCherry tagged lines, stained for α-synuclein (after stripping). Protein extract from rat brain with α-synuclein overexpressed and 293 T cells with mCherry overexpressed as positive controls. E: Full size Western Blot membrane for all the lines stained with the loading control Vinculin. Download Figure 2-1, TIF file. [file eneuro-12-ENEURO.0093-25.2025-s012.tif]

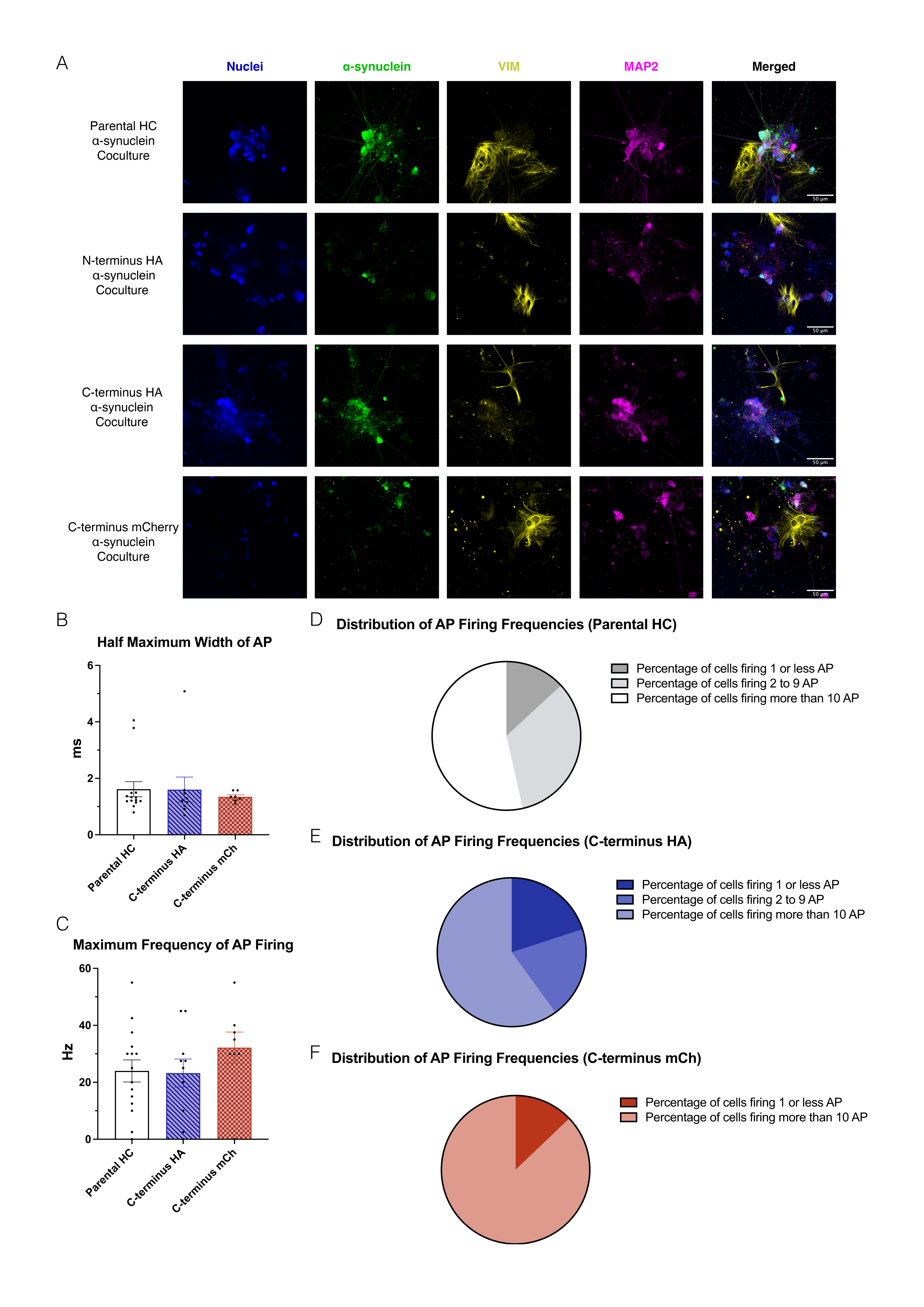

Supplement: Figure 3-1 — Additional electrophysiological data from patch clamp recordings of induced neurons from the different iPSC lines. A: Representative immunocytochemistry images of mature cocultures of iNs and iAs, positive for α-synuclein, neuronal marker MAP2 and astrocytic marker Vimentin (VIM). B: Half maximum width of action potentials of iNs from the different iPSC lines measured in ms. Bar graphs show the mean ± s.e.m. with n = 14 for the parental HC, n = 19 for C-terminus HA line, and n = 7 for C-terminus mCherry line. Scale bar = 50 μm. C: Maximum frequency of action potential firing of iNs from the different iPSC lines measured in Hz. Bar graphs show the mean ± s.e.m. with n = 15 for the parental HC, n = 10 for C-terminus HA line, and n = 8 for C-terminus mCherry line. D: Distribution of cells by action potential firing frequencies in the healthy control line. E: Distribution of cells by action potential firing frequencies in the C-terminus HA line. F: Distribution of cells by action potential firing frequencies in the C-terminus mCherry line. For B and C, one-way ANOVA was used to compare the means of the electrophysiological parameters across the groups of neurons. Assumptions of normality and homogeneity of variances were verified prior to analysis. A significance level of α = 0.05 was used and no statistical differences were detected. Download Figure 3-1, TIF file. [file eneuro-12-ENEURO.0093-25.2025-s013.tif]

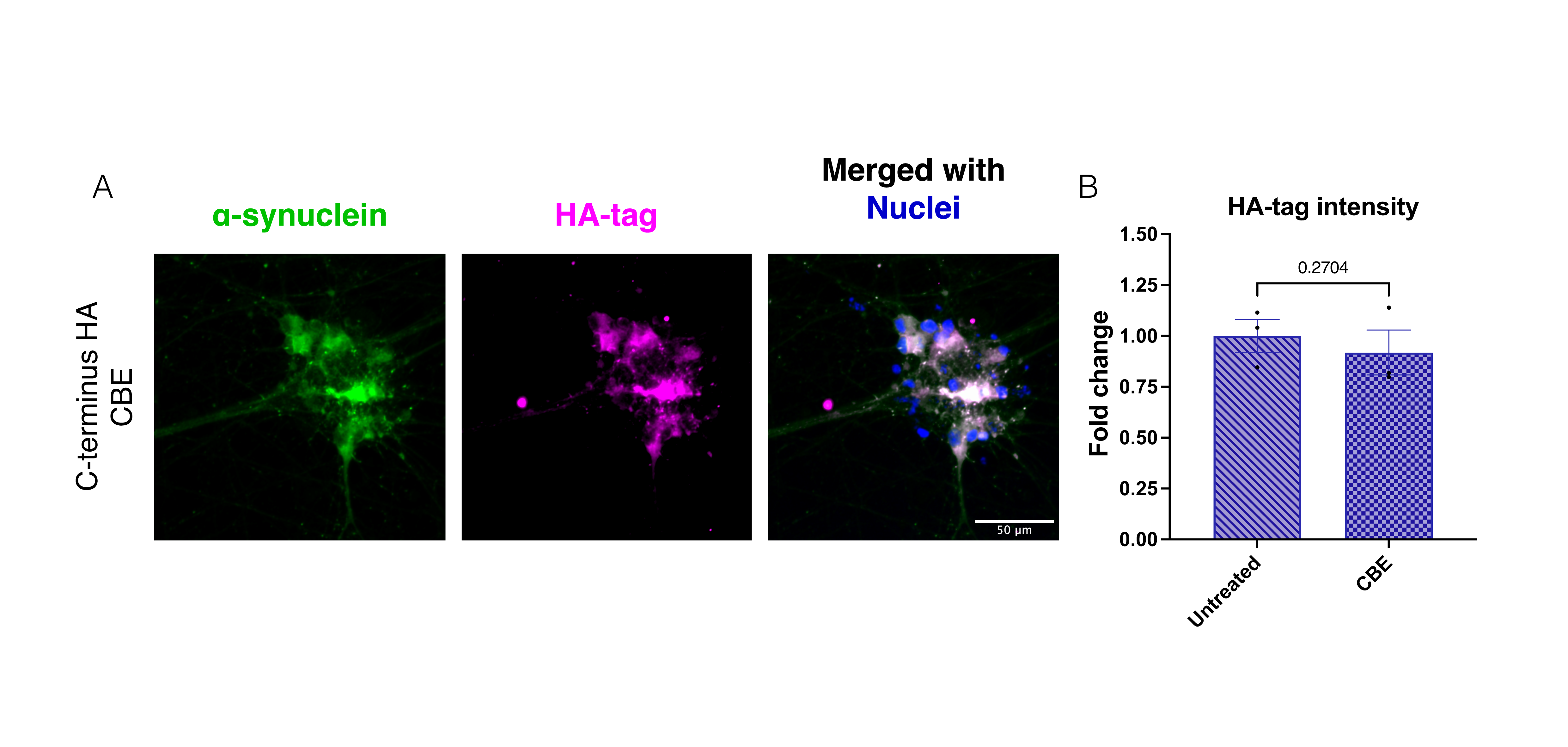

Supplement: Figure 4-1 — CBE treatment of cocultures from C-terminus HA-tagged line A: Representative immunocytochemistry images of mature cocultures of iN and iAs from the C-terminus HA-tagged iPSC line following treatment with CBE. Scale bar = 50 μm. B: Fold change of HA intensity in cocultures derived from the C-terminus HA-tagged iPSC line, comparing CBE treated to untreated cells (from Fig 4), and normalized within each experiment. Bar graph shows mean ± s.e.m. of 3 independent experiments. One-tailed ratio paired t-test was used to analyze the potential increase in intensity upon treatment, with a significance level of α = 0.05, and no significant difference was detected. Download Figure 4-1, TIF file. [file eneuro-12-ENEURO.0093-25.2025-s014.tif]
