# Supplementary material for: Generation of iPSC Lines with Tagged α-Synuclein for Visualization of Endogenous Protein in Human Cellular Models of Neurodegenerative Disorders
Source: eNeuro. 2025 Jun 10;12(6):ENEURO.0093-25.2025. doi: 10.1523/ENEURO.0093-25.2025 (PMC12186606; doi:10.1523/ENEURO.0093-25.2025)
Supplement: Figure 1-7 — Efficiency of the sgRNAs targeting the different regions of the SNCA gene. Each sgRNA was cloned into both the pX459 (wild-type Cas9) and the pX462 (nickase Cas9) plasmids. The pX462 plasmids were always used in combinations of sgRNAs targeting the sense and antisense strands. Download Figure 1-7, DOCX file. [file eneuro-12-ENEURO.0093-25.2025-s009.docx]

Figure 1-7: Efficiency of the sgRNAs targeting the different regions of the *SNCA* gene. Each sgRNA was cloned into both the pX459 (wild-type Cas9) and the pX462 (nickase Cas9) plasmids. The pX462 plasmids were always used in combinations of sgRNAs targeting the sense and antisense strands.

| **Target**  **terminus** | **Name** | **Number of confirmed alleles** | **Number of edited alleles** | **% of small indels***  **(< 20 bp)** | **% of editing efficiency** |
| --- | --- | --- | --- | --- | --- |
| pX459 | | | | | |
| N | sgRNA175 | 6 | 6 | 66.67 | 100 |
|  | sgRNA141 | 12 | 12 | 0 | 100 |
|  | sgRNA111 | 8 | 8 | 0 | 100 |
|  | sgRNA84 | 8 | 8 | 87.5 | 100 |
| C | sgRNA115 | 9 | 9 | 100 | 100 |
|  | sgRNA108 | 9 | 3 | 66.67 | 30 |
|  | sgRNA81 | 10 | 2 | 50 | 20 |
|  | sgRNA76 | 11 | 7 | 100 | 63.64 |
| pX462 | | | | | |
| N | sgRNA175+111 | 11 | 11 | 0 | 100 |
|  | sgRNA175+84 | 11 | 11 | 9.1 | 100 |
|  | sgRNA141+84 | 10 | 10 | 10 | 100 |
| C | sgRNA115+81 | 10 | 0 | 0 | 0 |
|  | sgRNA115+76 | 12 | 2 | 0 | 16.67 |
|  | sgRNA108+81 | 10 | 0 | 0 | 0 |
|  | sgRNA108+76 | 12 | 3 | 33.3 | 25 |
