# Supplementary material for: Generation of iPSC Lines with Tagged α-Synuclein for Visualization of Endogenous Protein in Human Cellular Models of Neurodegenerative Disorders
Source: eNeuro. 2025 Jun 10;12(6):ENEURO.0093-25.2025. doi: 10.1523/ENEURO.0093-25.2025 (PMC12186606; doi:10.1523/ENEURO.0093-25.2025)
Supplement: Figure 1-8 — Summary of the number of clones examined and identified for each tag and location of integration within the SNCA gene. Download Figure 1-8, DOC file. [file eneuro-12-ENEURO.0093-25.2025-s010.doc]

Figure 1-8: Summary of the number of clones examined and identified for each tag and location of integration within the *SNCA* gene.

| Tag | HA | HA | mCherry |
| --- | --- | --- | --- |
| Location of tag | N-terminus | C-terminus | C-terminus |
| sgRNA | sgRNA84 | sgRNA115 | sgRNA115 |
| Clones analyzed | 30 | 34 | 28 |
| Homozygous clones | 1 | 3 | 3 |
| Heterozygous clones | 0 | 0 | 2 |
| Editing Efficiency | 3,33%  2 alleles out of 60 | 8,82%  6 alleles out of 68 | 14,29%  8 alleles out of 56 |
